# Probing ligand conformation and net dimensionality in a series of tetraphenylethene-based metal–organic frameworks

**DOI:** 10.3389/fchem.2024.1396123

**Published:** 2024-04-25

**Authors:** Hannah M. Johnson, Matthew J. Hurlock, Monipak F. Lare, Lauren V. Forseth, Dylan M. Mosset, Jiahong Li, Qiang Zhang

**Affiliations:** ^1^ Department of Chemistry, Washington State University, Pullman, WA, United States; ^2^ Nanoscale Sciences Department, Sandia National Laboratories, Albuquerque, NM, United States; ^3^ Materials Science and Engineering Program, Washington State University, Pullman, WA, United States

**Keywords:** metal-organic framework, ligand conformation, tetraphenylethene, net dimensionality, solvent lability

## Abstract

Tetraphenylethene-based ligands with lowered symmetry are promising building blocks for the construction of novel luminescent metal–organic frameworks (MOFs). However, few examples have been reported, and predicting the ligand conformation and the dimensionality of the resulting MOF remains challenging. In order to uncover how synthetic conditions and accessible ligand conformations may affect the resulting MOF structure, four new MOF structures were synthesized under solvothermal conditions using the meta-coordinated tetraphenylethene-based ligand *m*-ETTC and paddlewheel SBUs composed of Co(II), Cu(II), and Zn(II). WSU-10 (WSU = Washington State University) is formed with either Zn or Cu comprising stacked psuedo-2D layers. The dimensionality of WSU-10 can be intentionally increased through the addition of pyrazine as a pillar ligand into the synthesis, forming the 3D structure WSU-11. The third structure, WSU-20, is formed by the combination of Zn or Co with *m*-ETTC and is intrinsically 3D without the use of a pillar ligand; interestingly, this is the result of a distortion in the paddlewheel SBU. Finally, Cu was also found to form a new structure (WSU-12), which displays an *m*-ETTC conformation unique from that found in the other isolated MOFs. Structural features are compared across the series and a mechanistic relationship between WSU-10 and -20 is proposed, providing insight into the factors that can encourage the generation of frameworks with increased dimensionality.

## 1 Introduction

Metal–organic frameworks (MOFs) stand out amongst coordination polymers, a type of inorganic-organic polymeric material, due to their unique properties such as permanent porosity, a high degree of structural diversity, large internal surface areas, and tunability (Zhou et al., 2012; Furukawa et al., 2013). These properties have enabled MOFs for use in a variety of applications including gas adsorption and separations (Zhao et al., 2018; Ding et al., 2019; Ma et al., 2020), catalysis (Jiao and Jiang, 2019; Xiao and Jiang, 2019), energy storage (Xu et al., 2017; Zhao et al., 2018), chemical sensors (Banerjee et al., 2014; Lustig et al., 2017; Wang et al., 2018; Pamei and Puzari, 2019), cancer therapy (Hu et al., 2023; Zhong et al., 2023), and lighting devices (Lustig and Li, 2018). MOFs are constructed from the self-assembly of multitopic organic ligands and metal ions or clusters called secondary building units, SBUs (Kalmutzki et al., 2018). The ligand selection plays a significant role in controlling the resulting structure and properties of the material. MOFs built from tetraphenylethene (TPE)-based molecules have attracted increased attention in the last decade due to their unique photoluminescent properties (Mei et al., 2015; Lustig et al., 2020; 2016; La et al., 2018; Hurlock et al., 2021a). TPE exhibits aggregation-induced emission (AIE) behavior, which can be mimicked through rigidification into a coordination scaffold, making it an ideal candidate for solid-state luminescent materials (Shustova et al., 2011; Wei et al., 2014; Lustig et al., 2019). Luminescent TPE-based MOFs are most commonly utilized in sensing applications, usually of contaminants or explosives (Liu et al., 2021), as well as solid-state emitters such as LEDs (Ma et al., 2017).

**FIGURE 1:**
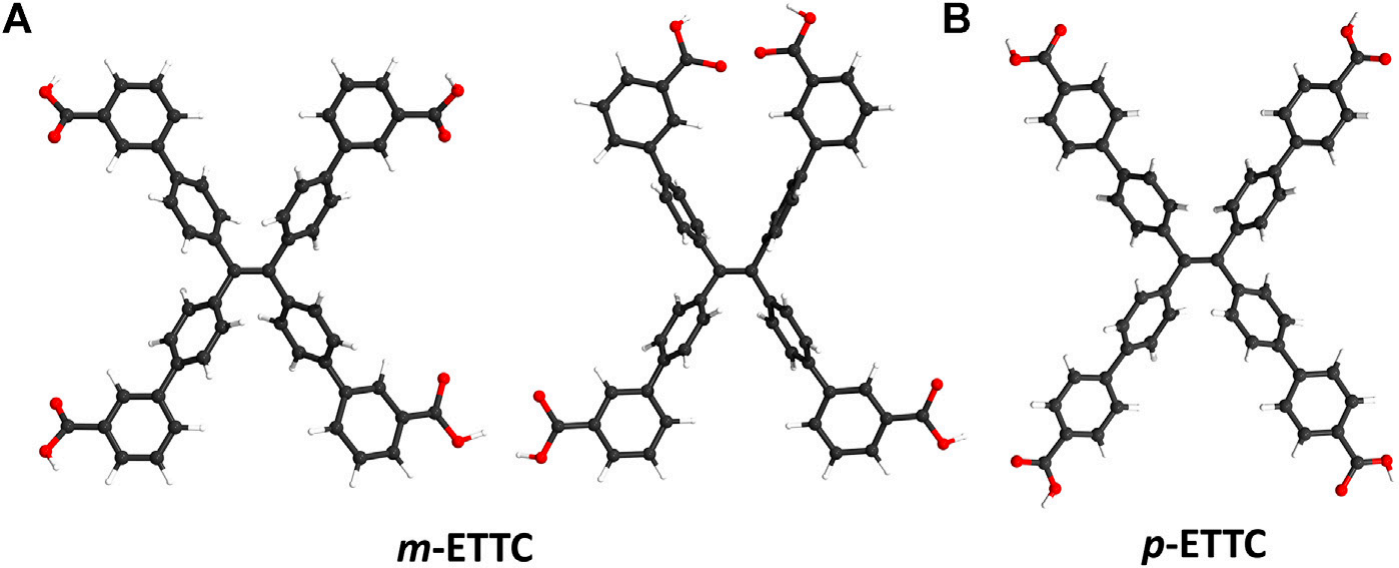
Illustrations of **(A)** two possible conformations of *m-*ETTC, compared with **(B)** *p*-ETTC (Atom representations: carbon is black, oxygen is red, and hydrogen is white).

The majority of TPE-based ligands used in fluorescent MOFs contain binding moieties in the para position of the end phenyl rings (Gong et al., 2014; Wei et al., 2014; Zhang et al., 2015; Yang et al., 2016), due in part to the ease of synthesis and purification of these compounds. In contrast, TPE-based ligands of lowered symmetry have seen little research interest, despite their potential for accessing more varied conformations and MOF structures without detriment to their photophysical properties. For example, the ligand 4′,4‴,4‴′,4‴‴′-(ethene-1,1,2,2-tetrayl)tetrakis([1,1′-biphenyl]-3-carboxylic acid) (*m-*ETTC) has a single binding moiety in the meta-position at the end of each phenyl arm. This results in a wide range of conformations the ligand can adopt due to the rotation of the terminal phenyl rings, seen in Figure 1A. For comparison, the tetratopic para-ETTC (*p*-ETTC) ligand (Figure 1B) is generally considered a simple rectangular or square node in MOFs. Predicting the conformation that ligands like *m-*ETTC will adopt *a priori* in solid-state structures is difficult, as it is highly dependent on the synthetic conditions and the geometry of the metal nodes.

Fortunately, predicting and influencing the geometry of the metal SBU is simpler compared to ligand conformation. For example, divalent transition metals like Zn(II), Cu(II), and Co(II) commonly form paddlewheel clusters when combined with carboxylate ligands (Mori et al., 2005; Carson et al., 2009). Paddlewheel SBUs typically coordinate to four carboxylate groups, whose conformation with respect to the linker influences the resulting dimensionality of the MOF (Furukawa et al., 2008). In literature so far, the combination of paddlewheel clusters and para-coordinated TPE-based linkers (namely, ETTC and TCPE) has only been found to form layered 2D sheet structures (Shustova et al., 2011; King et al., 2016; Zheng et al., 2020). In comparison, *m-*ETTC has a higher potential to form 3D frameworks with paddlewheel SBUs due to the increased diversity of its conformations.

To examine what synthetic factors may affect the conformation of the *m-*ETTC ligand and overall framework dimensionality, we have synthesized four distinct paddlewheel-MOF structures using *m-*ETTC and divalent metal cations. The first MOF, WSU-10 (WSU = Washington State University), possesses a pseudo 2D sheet structure with the formula M_2_(*m-*ETTC) (H_2_O)_2_ (M = Cu or Zn). Upon addition of pyrazine as a pillar ligand to the reaction mixture, a new 3D structure denoted WSU-11 is formed, or M_2_(*m-*ETTC) (½pyz) (H_2_O) (M = Cu or Zn, pyz = pyrazine). A third structure was identified and determined to be intrinsically 3D, named WSU-12 or M_2_(*m-*ETTC) (DMAc)_2_ (M = Cu, DMAc = dimethylacetamide), and a final 3D structure named WSU-20 was isolated and found to be M_2_(*m-*ETTC) (DMAc)_2_ (M = Co or Zn). The bulk properties of these materials were assessed, and structural variations between the compounds resulting from changes to the synthetic conditions have been characterized. Additionally, broad trends in node geometry, ligand conformation, and net dimensionality are presented.

## 2 Materials and methods

### 2.1 Chemicals

Copper nitrate hemi-pentahydrate (99%), bromine, and tetraphenylethene (TPE, 98%) were purchased from Alfa Aesar. Cobalt nitrate hexahydrate was purchased from J. T. Baker. Zinc nitrate hexahydrate (99%) was purchased from Beantown Chemical. Nitric acid and tetrahydrofuran were purchased from Fisher Scientific. *N,N*-dimethylformamide (DMF) was purchased from MilliporeSigma, while *N,N*-dimethylacetamide (DMAc), potassium hydroxide, *p*-dioxane, dichloromethane (DCM), ethanol, and methanol were purchased from EMD Millipore. (3-(methoxycarbonyl)phenyl)boronic acid (95%) was purchased from Oxchem. Cesium fluoride and pyrazine were purchased from Oakwood Chemical. Tetrakis(triphenylphosphine)palladium(0) (99%) was purchased from Strem Chemicals. Nanopure water was made using a Barstead Nanopure with a D5026 organic free filter kit. All chemicals were used as received without further purification.

### 2.2 Synthetic procedure

#### 2.2.1 Synthesis of 4′,4‴,4‴′,4‴‴′-(ethene-1,1,2,2-tetrayl)tetrakis (([1,1′-biphenyl]-3-carboxylic acid)) (*m-*H_4_ETTC)

*m-*H_4_ETTC was synthesized following previously reported procedures (Hurlock et al., 2021b).

#### 2.2.2 Preparation of WSU-10(Cu)

*m-*H_4_ETTC (30 mg, 0.037 mmol) and Cu(NO_3_)_2_·2.5H_2_O (90 mg, 0.38 mmol, 10.5 equiv.) were dissolved in DMAc (2 mL) in a 4 mL glass vial, followed by 200 µL of concentrated HNO_3_, giving a transparent green solution. The vial was shaken vigorously before being placed in an oven at 100°C. After 48 h, green square plate crystals had formed, and the vial was removed from the oven. After allowing the vial to cool, quality crystals were collected for structure determination. The remaining crystals were washed with DMF (3 × 4 mL) and soaked for 24 h in fresh DMF. The crystals were washed with methanol (3 × 4 mL) and soaked in fresh methanol for 4 days, exchanging with fresh methanol every day. The methanol was then decanted, and the crystals were dried at room temperature under vacuum for 5 h giving the product as a green crystalline powder (23.1 mg, 64.5% yield based on *m-*H_4_ETTC).

#### 2.2.3 Preparation of WSU-10(Zn)

*m-*H_4_ETTC (30 mg, 0.037 mmol) and Zn(NO_3_)_2_·6H_2_O (90 mg, 0.372 mmol, 10 equiv.) were dissolved in DMAc (4 mL) in a 20 mL glass vial, followed by 500 µL of Nanopure water, giving a transparent pale-yellow solution. The vial was shaken vigorously before being placed in an oven at 85°C. After 1 h, the vial was removed from the oven and allowed to cool. After cooling, yellow plate crystals had formed. Quality crystals were collected for structure determination. The remaining crystals were then washed with DMF (3 × 8 mL) and soaked for 24 h in fresh DMF. The crystals were washed with methanol (3 × 8 mL) and soaked in fresh methanol for 4 days, exchanging with fresh methanol every day. The methanol was then decanted, and the crystals were dried at room temperature under vacuum for 16 h giving the product as a yellow crystalline powder (Yield: 9.3 mg, 25.8% yield based on *m-*H_4_ETTC).

#### 2.2.4 Preparation of WSU-11(Cu)

The following modifications were made to the synthesis of WSU-10(Cu) to synthesize WSU-11(Cu): pyrazine (5 mg, 0.0625 mmol, 1.67 equiv.) was added, and the total volume of DMAc was changed to 3 mL. (Yield: 31.1 mg, 84.5% based on *m-*H_4_ETTC).

#### 2.2.5 Preparation of WSU-11(Zn)

The following modifications were made to the synthesis of WSU-10(Zn) to synthesize WSU-11(Zn): pyrazine (5 mg, 0.0625 mmol, 1.67 equiv.) was added to the reaction solution before heating. (Yield: 9.7 mg, 26.4% based on *m-*H_4_ETTC).

#### 2.2.6 Preparation of WSU-20(Co)

*m-*H_4_ETTC (60 mg, 0.074 mmol) and Co(NO_3_)_2_·6H_2_O (30 mg, 0.103 mmol, 0.72 equiv.) were dissolved in DMAc (8 mL) in a 20 mL glass vial, followed by 200 µL of Nanopure water, giving a deep purple solution. The vial was shaken vigorously before being placed in an oven at 100°C. After 48 h, purple block crystals had formed, and the vial was removed from the oven. After allowing the vial to cool, quality crystals were collected for structure determination. The remaining crystals were then washed with DMAc (3 × 4 mL) and soaked for 24 h in fresh DMAc. The crystals were washed with DCM (3 × 4 mL) and soaked in fresh DCM for 4 days, exchanging with fresh DCM every day. The DCM was then decanted, and the crystals were dried at room temperature in air for 16 h giving the product as a purple-red crystalline powder (Yield: 23.4 mg, 30.6% yield based on *m-*H_4_ETTC).

#### 2.2.7 Preparation of WSU-20(Zn)

*m-*H_4_ETTC (30 mg, 0.037 mmol) and Zn(NO_3_)_2_·6H_2_O (90 mg, 0.372 mmol, 10 equiv.) were dissolved in DMAc (4 mL) in a 20 mL glass vial, giving a transparent yellow solution. The vial was shaken vigorously before being placed on a hot plate at 40°C. After 72 h, yellow pillar crystals had formed, and the vial was removed from the hot plate. After allowing the vial to cool, quality crystals were collected for structure determination. The remaining crystals were then washed with DMF (3 × 4 mL) and soaked for 24 h in fresh DMF. The crystals were washed with DCM (3 × 4 mL) and soaked in fresh DCM for 4 days, exchanging with fresh DCM every day. The DCM was then decanted, and the crystals were dried at room temperature in air for 16 h giving the product as a yellow crystalline powder (Yield: 4.0 mg, 9.7% yield based on *m-*H_4_ETTC).

### 2.3 Instrumentation

Powder X-ray diffraction (PXRD) patterns were collected using a Rigaku MiniFlex600 diffractometer equipped with a Cu K_α_ source (λ = 1.5406 Å). The generator power was set at 40 kV and 15 mA. The data was collected in the 2θ range of 5°–40°, with a step size of 0.02° and a scan speed of 4° min^-1^. Photoluminescence emission spectra were obtained at room temperature using a Horiba FluoroMax-4 equipped with a Xenon lamp and an excitation wavelength of 365 nm. Optical microscopy images were obtained on glass slides using a Leica M165 C microscope with an M170 HD camera. SEM samples were sputter-coated with gold using a Technics Hummer V Sputter Coater and images were obtained using a FEI SEM Quanta 200F at 20 kV. UV-Vis spectra were obtained using a ThermoScientific Evolution 300 UV/Vis spectrophotometer using a diffuse reflectance Harrick Praying Mantis attachment and a Harrick Sampling kit DRP-SAP. Fourier transform infrared (FTIR) spectra were collected using a ThermoScientific Nicolet iS10 with the iTR solid-state accessory.

### 2.4 Single crystal X-Ray diffraction (SCXRD)

High-quality single crystals were selected from reaction mixtures using an optical microscope and placed onto MiTiGen Dual Thickness MicroMounts using paratone oil. Single crystal data were collected at room temperature on a Bruker D8 Venture with a microfocus source and Mo K_α_ radiation (λ = 0.71073 Å). Absorption corrections were done using the SADABS (Dolomanov et al., 2009) detector absorption correction program embedded in APEX3 (Adam et al., 2015). The structures were solved using SHELXT (Sheldrick, 2015a) structure solution program using Intrinsic Phasing, and refinement was done using SHELXL (Sheldrick, 2015b) refinement package and least-squares minimization embedded into the Olex2 interface (Bourhis et al., 2015). All non-hydrogen atoms were refined with anisotropic thermal parameters. Hydrogen atoms were located by using difference Fourier maps and were placed in the geometrically calculated positions and refined using a riding model. The solvent masking feature of Olex2 was used on all structures. Detailed crystallographic data and refinement parameters are summarized in Supplementary Table S1.

## 3 Results

### 3.1 Structural description

The TPE-based ligand *m-*ETTC was synthesized following previously reported procedures (Hurlock et al., 2021b). Using *m-*ETTC and metal nitrate precursors of Zn, Cu, and Co, a series of coordination polymers were synthesized through individually optimized solvothermal methods. Single crystals of the seven compounds were obtained, Supplementary Figure S1, and single-crystal X-ray diffraction (SCXRD) was used to determine their structures. A summary of the crystallographic data is provided in Supplementary Table S1, and the structure of WSU-10(Zn) has been reported previously (Wang et al., 2019; Xu et al., 2022).

#### 3.1.1 Structure of WSU-10

**FIGURE 2:**
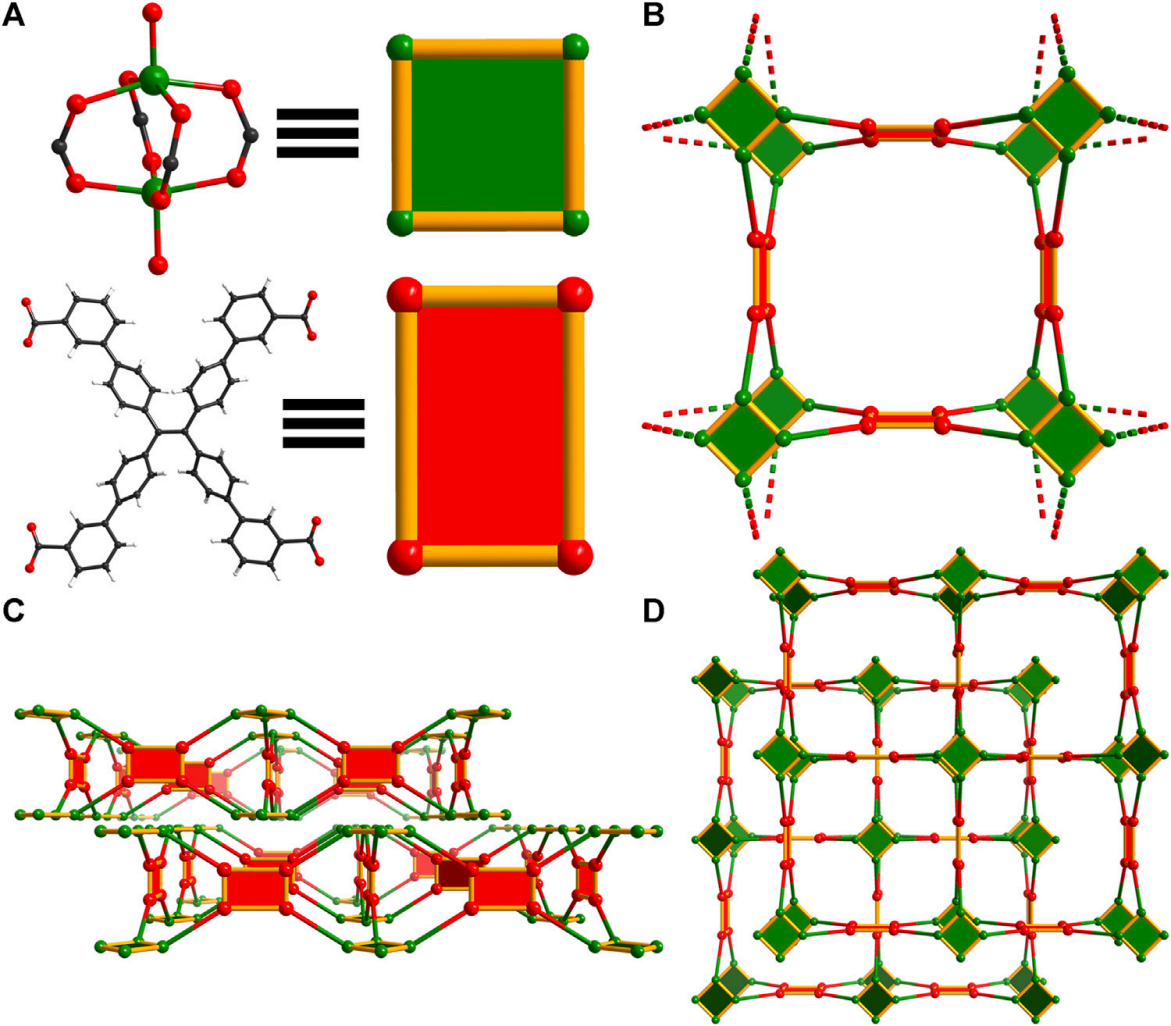
Topology illustration of the structure of WSU-10(Cu). **(A)** The paddlewheel Cu_2_ cluster in WSU-10(Cu) and the conformation of *m-*ETTC ligands representing square nodes; **(B)** depiction of the view of one unit in WSU-10(Cu), as viewed along the c-axis; **(C)** depiction of two layers, as viewed along the a-axis, and **(D)** depiction of the stacking of two layers in WSU-10(Cu), as viewed along the c-axis.

To form WSU-10(Cu), copper(II) nitrate and *m-*H_4_ETTC were combined in *N,N*-dimethylacetamide (DMAc) with nitric acid, yielding the product as large green square plates, Supplementary Figure S1A. It was found that the typical solvent *N,N*-dimethylformamide (DMF) cannot be used as Cu(II) is reduced, depositing copper metal along the vial walls. WSU-10(Cu) crystallizes in the tetragonal space group *P*4*/nnc*, and the asymmetric unit contains one-quarter of the *m-*ETTC ligand and two independent Cu(II) ions. These ions are penta-coordinated, involving four oxygen atoms of different *m-*ETTC ligands and one capping oxygen from a coordinating water molecule, forming the paddlewheel SBU (Figure 2A and Supplementary Figure S2A). The *m-*ETTC ligand orients all carboxylate groups outward, approximately within the plane of the ethene core. This orientation of the ligand, in combination with the copper paddlewheel cluster, leads to a single pseudo-2D layer with the thickness of an *m-*ETTC ligand, Figure 2B and Supplementary Figure S2B. These layers stack together in an ABA fashion to form the packed arrangement of WSU-10, Figures 2C, D, Supplementary Figures S2C, E. Due to the staggering of the layers, the pores within each layer are occupied by the clusters of the adjacent layer. Its topology was analyzed using Topcryst (Shevchenko et al., 2022) and can be described as a 4,4-connected (for the ligand and metal node, respectively) pseudo-two dimensional structure, Figure 2B, possessing the underlying topology **4,4L1**.

An isostructural Zn analogue of WSU-10(Cu) was obtained by replacing the metal precursor, using water in place of nitric acid, and reducing the reaction temperature and time. Though WSU-10(Cu) is the first report of a Cu-ETTC MOF, there have been previous reports of the WSU-10(Zn) structure (Wang et al., 2019; Xu et al., 2022). These describe nearly identical MOFs of Zn_2_ paddlewheels and pseudo-2D *m-*ETTC layers; interestingly, in the 2019 report, adjacent layers are connected to each other through bridging μ_2_-CO_2_ molecules that were determined to have been acquired from the ambient air atmosphere. This resulted in new connectivity (a 2-fold interpenetrated 3D net) but otherwise the same spatial arrangement of atoms that is found in the 2D MOFs presented here.

#### 3.1.2 Structure of WSU-12

**FIGURE 3:**
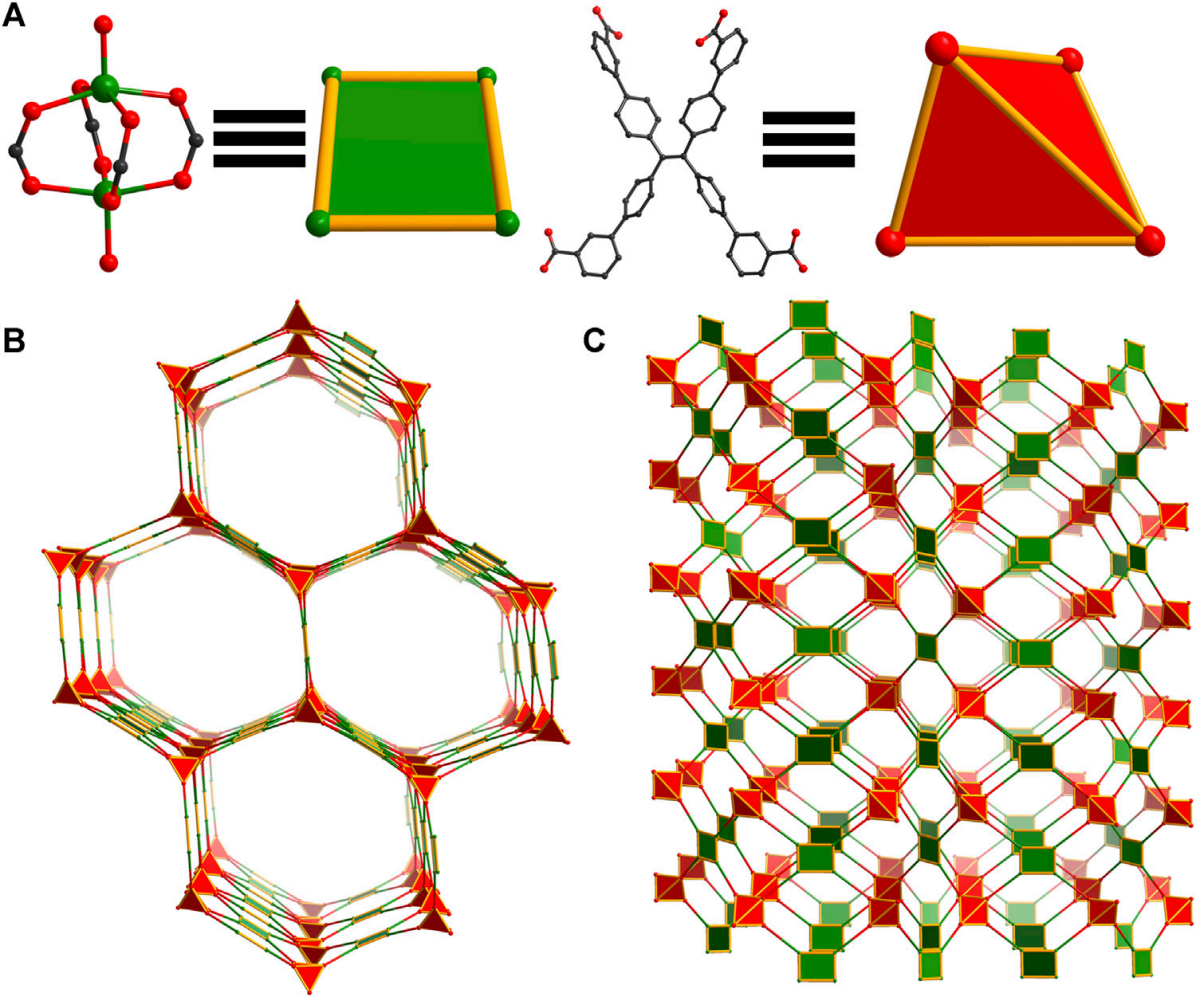
Topology illustration of the structure of WSU-12(Cu). **(A)** The paddlewheel Cu_2_ cluster in WSU-12(Cu) representing square nodes and the conformation of *m-*ETTC ligands representing tetrahedral nodes; **(B)** depiction of the view of WSU-12(Cu), as viewed along the [101] direction; **(C)** depiction of a single net of the structure, as viewed along the b-axis.

A second population of crystals was identified in reactions of WSU-10(Cu) due to slight differences in morphology (elongated hexagonal plates, Supplementary Figure S3). SCXRD determined that this phase is in fact a unique 3D structure in the *C*2*/c* space group, denoted WSU-12(Cu). Featuring similar paddlewheel nodes that are DMAc-capped (Figure 3A and Supplementary Figure S4A), this 3D structure arises from a difference in orientation of *m-*ETTC, wherein two carboxylates have twisted out of the plane of the ethene core in opposite directions, Figure 3A and Supplementary Figure S4B, forming a pseudo tetrahedral conformation, Figure 3A. This conformation change breaks the layered structure observed in WSU-10(Cu) and instead promotes an intrinsically 3D structure with **4,4,4T72** topology, Figure 3C, featuring large channel pores (24 × 32 Å, Figure 3B and Supplementary Figure S5A). WSU-12 also possesses a 4,4-connected net; however, it is 3D instead of 2D. This also causes independent nets of WSU-12(Cu) to assemble within each other, resulting in a 3-fold interpenetrated structure (Supplementary Figure S5B) that retains channels with a diagonal width of 16 Å. Attempts to isolate this structure as a pure phase were unsuccessful and further physical characterization was not performed, though its structure is discussed in following sections.

#### 3.1.3 Structure of WSU-11

**FIGURE 4:**
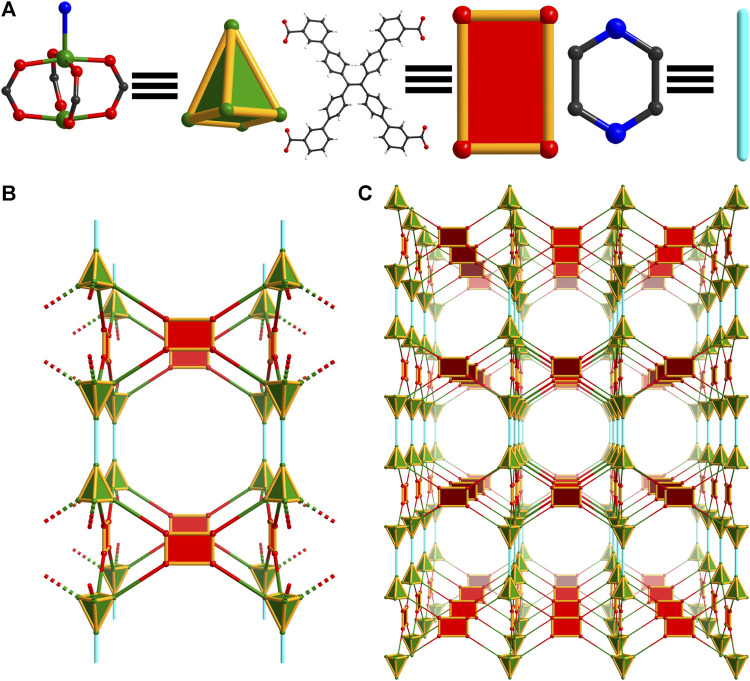
Topology illustration of the structure of WSU-11(Cu). **(A)** The paddlewheel Cu_2_ cluster in WSU-11(Cu) with the axial ligand representing a square pyramid node, and the conformation of *m-*ETTC ligands representing rectangular nodes; **(B)** depiction of a single net of the structure showing two layers being linked by the pyrazine pillar, as viewed along the b-axis. **(C)** Depiction of a single net of the structure showing expanded structure, as viewed along the b-axis.

Though residual electron density suggests that carbon dioxide is present in the structures of WSU-10(Cu) and WSU-10(Zn), similar to previous reports (Wang et al., 2019), this cannot be well-resolved due to disorder/low occupancy (Supplementary Figure S6). In order to clearly target this bridging connection and increase the dimensionality of the framework, pyrazine was selected as a substitute pillar molecule due to its similarity in size and coordination mode to CO_2_. The introduction of pyrazine into the synthesis of WSU-10(Cu) resulted in crystals with a square plate morphology, Supplementary Figure S1B, which successfully incorporated pyrazine pillars between layers of WSU-10. The new MOF, WSU-11(Cu), crystallizes in the same tetragonal space group *P*4*/nnc*, and its unit cell parameters are almost identical to that of WSU-10(Cu) except for an elongation of the *c*-axis caused by the incorporation of pyrazine (Supplementary Table S1). Much like WSU-10(Cu), the metal centers of WSU-11(Cu) are penta-coordinated (Figure 4A and Supplementary Figure S7A), though the Cu atoms on the exterior of the pseudo-2D layers are coordinated to a nitrogen of pyrazine molecules which bridge two paddlewheel clusters from two different layers (Figure 4B and Supplementary Figure S7B). This bridging connection causes the structure to transform from 2D to a 3D structure with 2-fold interpenetration, Supplementary Figures S7C, D. The underlying net of WSU-11(Cu) can be described as 4,5-connected with the topology **xah,** Figure 4C. The use of larger molecules (such as 4,4′-bipyridine) to expand the layers was explored; however, no crystals containing these molecules were obtained. The isostructural WSU-11(Zn) was obtained by similarly adding pyrazine to the synthesis of WSU-10(Zn), mimicking the connectivity of the CO_2_-bridged 3D structure described previously.

#### 3.1.4 Structure of WSU-20

**FIGURE 5:**
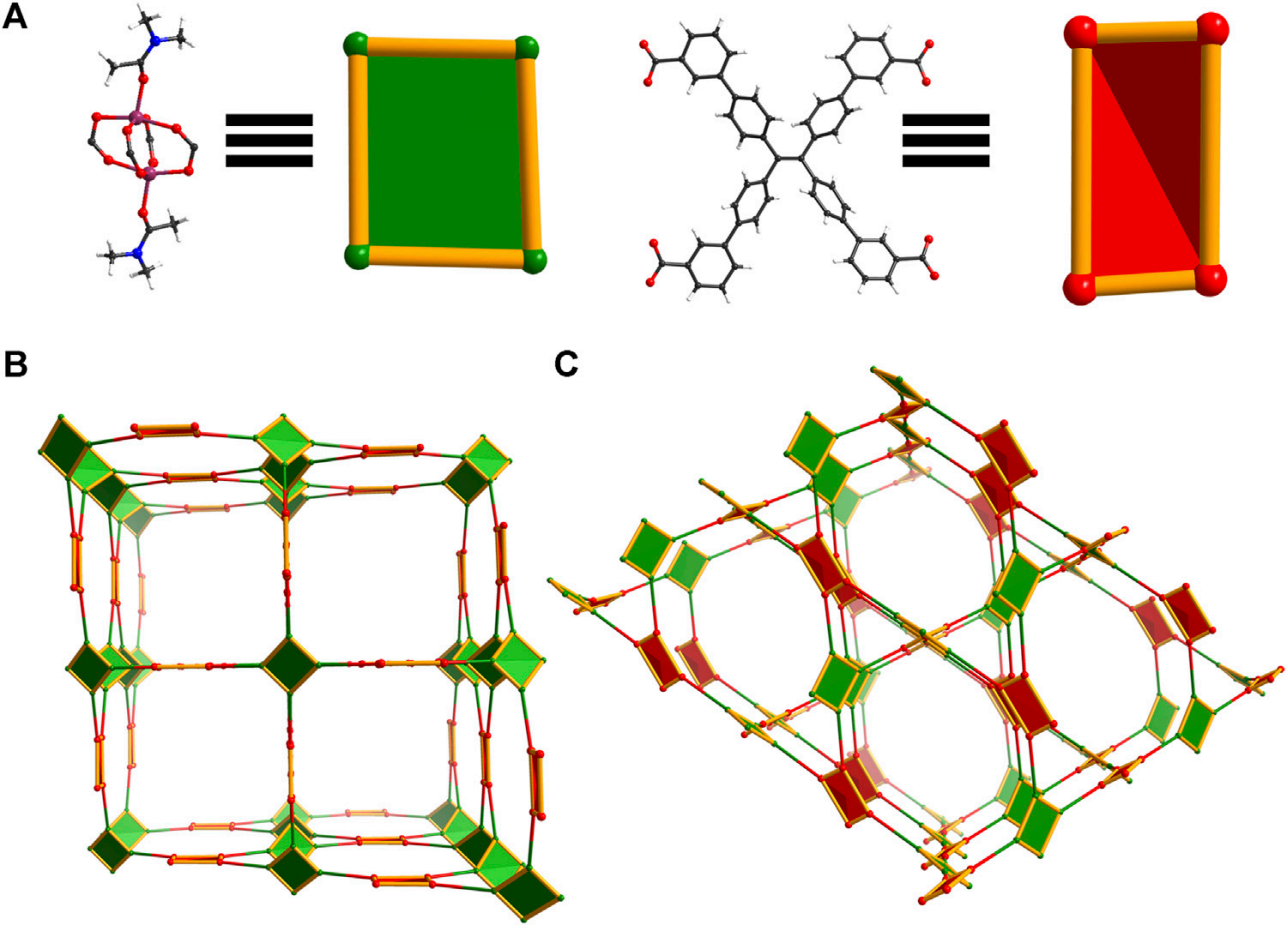
Topology illustration of the structure of WSU-20(Co). **(A)** The distorted paddlewheel Co_2_ cluster in WSU-20(Co) representing a distorted rectangular node, and the conformation of *m-*ETTC ligands representing a pseudo tetrahedral node; **(B)** depiction of the structure, as viewed along the c-axis; **(C)** depiction of the structure, as viewed along the [ $1\bar{1}0$ ] direction.

Through further synthetic manipulation of the WSU-10 synthesis, another structure was achieved featuring *m-*ETTC and paddlewheel clusters. Cobalt(II) nitrate was used as the metal precursor, the reaction was made more dilute, and water was used in place of nitric acid as the modulator. Under these conditions using DMAc as the solvent, red-violet rod-shaped crystals were obtained (Supplementary Figure S1D). The structure, named WSU-20(Co), crystallizes in the monoclinic space group *C*2*/c*. The asymmetric unit contains one *m-*ETTC molecule, two independent Co atoms, and two disordered coordinated DMAc molecules. Each Co(II) ion is penta-coordinated with four oxygen atoms originating from four *m-*ETTC ligands and one from the DMAc cap, Figure 5A and Supplementary Figure S8A. The paddlewheel cluster of WSU-20 is distorted compared to that of WSU-10 and -11, caused by the steric interactions of the coordinated DMAc molecules between the stacked SBUs. The two sides of the paddlewheel ‘slip’ to avoid direct DMAc overlap, preventing the propagation of a single layer with aligned linkers. Instead, the *m-*ETTC molecules are arranged in a checkerboard fashion, forming an intrinsically 4,4-connected 3D net with **lvt** topology, Figures 5B, C and Supplementary Figure S8B, that is 2-fold interpenetrated, Supplementary Figures S8C, D. The isostructural compound WSU-20(Zn) was obtained by reducing the reaction temperature from 100°C to 40°C. Above this temperature, mixed-phase products of WSU-10(Zn) and WSU-20(Zn) result, indicating a kinetic preference for the WSU-20(Zn) phase *versus* a thermodynamic preference for WSU-10(Zn). A Cu analogue of WSU-20 was pursued but was not successful, as all synthesis attempts resulted in WSU-10(Cu).

### 3.2 Assessment of properties

**FIGURE 6:**
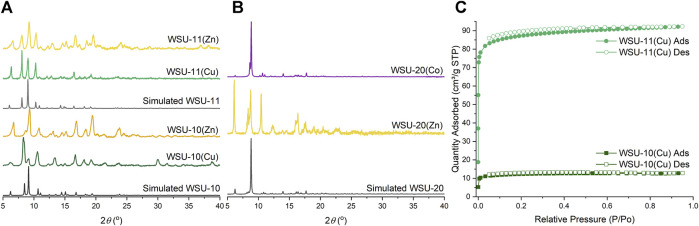
**(A)** Powder XRD patterns of simulated WSU-10 and WSU-11 (black), WSU-10(Cu) and WSU-11(Cu) (green), and WSU-10(Zn) and WSU-11(Zn) (yellow). **(B)** Powder XRD patterns of simulated WSU-20 (black), WSU-20(Zn) (yellow), and WSU-20(Co) (purple). **(C)** N_2_ gas adsorption isotherms at 77K of WSU-10(Cu) (dark green squares) and WSU-11(Cu) (light green circles).

Infrared spectra (Supplementary Figure S9) confirm the presence of the *m*-ETTC ligand and the successful coordination of the carboxylate groups in bulk material. To determine phase purity of all reactions, powder X-ray diffraction (PXRD) patterns on bulk material were collected, Figures 6A, B. Due to the subtle differences between the structures and lattice parameters of WSU-10 and WSU-11, their crystal morphologies and diffraction patterns are quite similar. The two compounds can be best distinguished by the positions of reflections below 12° (2θ) (Supplementary Figure S10), as the insertion of the pyrazine ligand increases the interlayer spacing and decreases the position of reflection. The variations between the experimental and simulated patterns of WSU-10 are likely due to facile changes in the interlayer spacing caused by sample processing and preparation. This is not observed in the patterns of WSU-11, as the pyrazine locks the layers in fixed positions. Additionally, the pattern of the WSU-10(Cu) does not match any major peaks exhibited by the simulated structure of WSU-12(Cu) (Supplementary Figure S11), confirming that WSU-12(Cu) constitutes only a minor impurity in reactions of WSU-10(Cu).

The effects of pyrazine insertion on the MOFs’ gas uptake properties were examined with nitrogen adsorption experiments at 77K. The compound WSU-10(Cu) shows microporous adsorption with an N_2_ uptake of 14 cm^3^/g and a low Brunauer–Emmett–Teller (BET) surface area of 46 m^2^/g, Figure 6C. When pyrazine is introduced into the structure in WSU-11(Cu), N_2_ adsorption increases to 92 cm^3^/g with a corresponding rise in the BET surface area to 335 m^2^/g, a seven-fold increase from WSU-10(Cu). The experimental pore size distributions similarly reflect a cohesive increase in accessible pore space (Supplementary Figure S12). The non-copper compounds WSU-10(Zn), WSU-11(Zn), and WSU-20(Zn/Co) exhibited poor stabilities under heating at low pressures, forming non-porous amorphous solids and precluding proper activation and N_2_ adsorption analysis. In the case of WSU-20, the removal of the bulky solvent caps at the metal centers during solvent exchange likely alters the distorted nature of the paddlewheel, straining the framework and ultimately leading to structural collapse. Low stability is not uncommon for other reported paddlewheel-based frameworks (Tan et al., 2012).

Optical absorption spectra of all compounds were obtained at room temperature, Supplementary Figure S13A. WSU-10(Zn), −11(Zn), −20(Zn) are all yellow in color under ambient light and show absorbance spectra similar to the free *m-*H_4_ETTC ligand. The compounds WSU-10(Cu) and −11(Cu) show an absorption with a maximum of 675 nm, due to the copper metal centers, resulting in the green color of the compound. The as-synthesized WSU-20(Co) compound exhibits a deep violet color due to the absorbance centered at 550 nm by cobalt, though this color was observed to vary with solvent. Structural degradation of WSU-20 upon solvent exchange hindered further study of this phenomenon. Furthermore, while the Cu(II)- and Co(II)-based compounds were found to be fluorescently nonemissive, the closed-shell *d* ^*10*^ compounds WSU-10(Zn), WSU-11(Zn), and WSU-20(Zn) all demonstrate strong fluorescence with λ_em_ at 508 nm, 509 nm, and 518 nm respectively when excited with UV light (Supplementary Figure S13B). These signals originate from the luminescent ligand, which emits at 510 nm, and do not deviate much between Zn-MOFs due to the similar conformation *m-*ETTC adopts across all three structures. It is interesting to note that WSU-20(Zn) exhibits the lowest energy emission, since WSU-20 also possesses a higher packing density than the other two structures. It has been previously observed that smaller interligand distances increase the likelihood of charge transfer and excimer formation between ligands, and interpenetrated structures tend to display redshifted emissions compared to their free fluorophore (Meek et al., 2010). The photophysical properties of the WSU-10(Zn) structure have also been investigated in its respective previous reports (Wang et al., 2019; Xu et al., 2022) and align well with the behavior observed here. Xu *et al.* also utilized the material’s layered structure to generate nanosheet suspensions, which were then applied toward the sensing of aqueous antibiotics via fluorescent quenching at low concentrations.

## 4 Discussion

**FIGURE 7:**
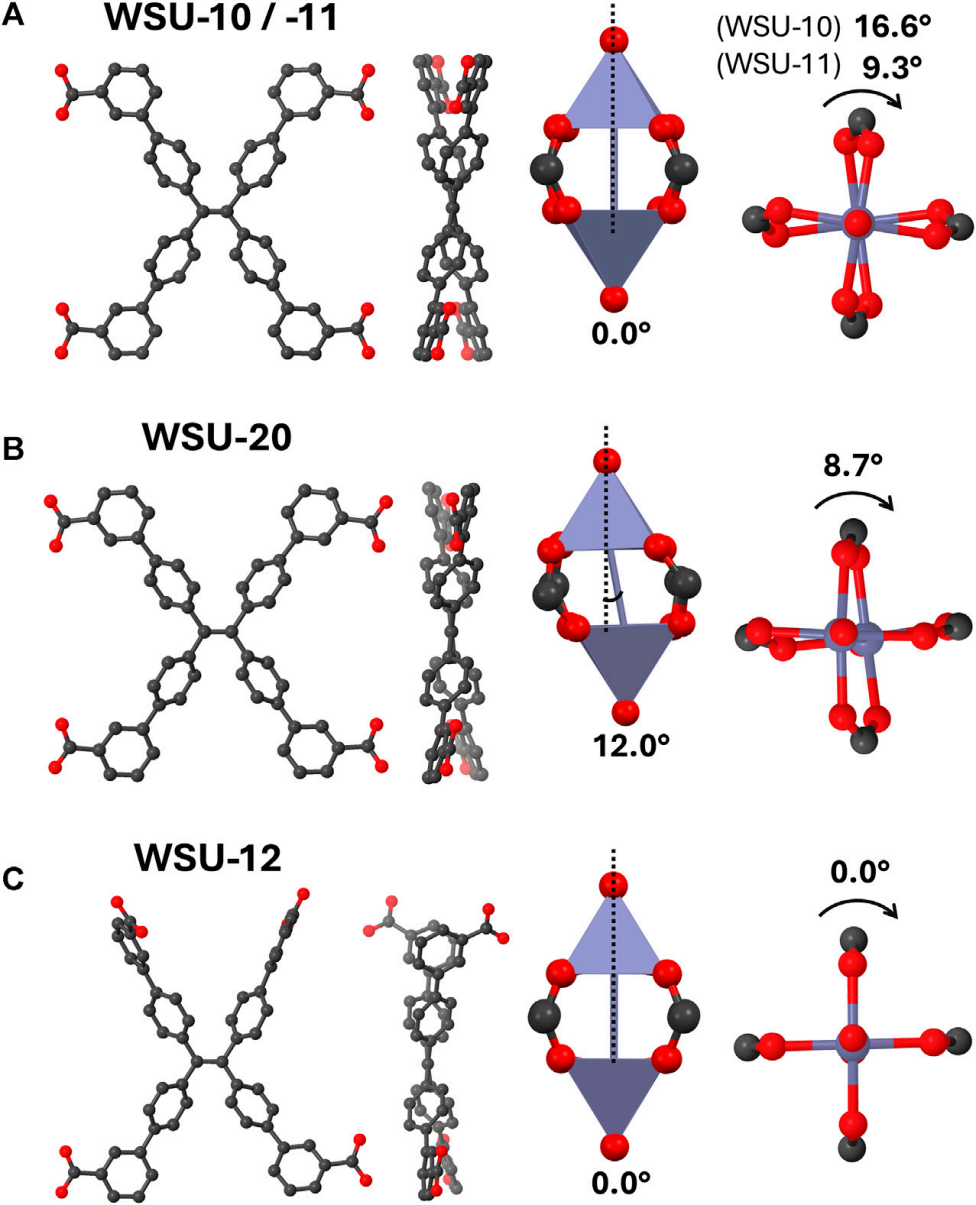
Diagrams of the ligand and node geometries seen in **(A)** WSU-10/-11, **(B)** WSU-20, and **(C)** WSU-12. (Atom representations: metals are purple, carbon is black, and oxygen is red; hydrogens omitted).

All MOFs reported herein are composed of the *m-*ETTC ligand and paddlewheel nodes, but vary in distinct ways that may give insight into the relationship between ligand/node geometry and overall framework structure. Note that conformations between isostructural samples of different elements generally varied by less than 1°. The orientations of *m-*ETTC in WSU-10 and -11 are essentially identical and will be treated together, demonstrating a symmetric conformation with all four carboxyl groups relatively in-plane with the ethene bond and pointing outwards (Figure 7A). This conformation has been previously observed in the only other two reported paddlewheel-*m-*ETTC MOFs, which share the WSU-10 structure (Wang et al., 2019; Xu et al., 2022). The pseudo-2D layered framework seems to be favored by this symmetric *m-*ETTC conformation and the square planar coordination motif of paddlewheel nodes. However, this ligand orientation is also found in WSU-20, with slightly different torsion angles due to the imprecise nature of the paddlewheel distortion (Figure 7B). WSU-20 represents the first time a 3D MOF has resulted from paddlewheel nodes and *m-*ETTC, and it is surprising that this occurred without a change in ligand orientation (*vide infra*). Additionally, a unique *m-*ETTC conformation is observed in WSU-12(Cu) which has not been reported in a MOF to date (Figure 7C). Two carboxyl groups on the same side of the central double bond have twisted out-of-plane and sit approximately perpendicularly to the plane of the ethene core, allowing subsequent paddlewheel coordination that extends in all axes and forms a 3D framework.

Turning to the configuration of the paddlewheel nodes, standard geometries are observed in WSU-10, -11, and −12 where the metal-metal distance is perpendicular to the coordination plane around the metals. The two square pyramids of the cluster are twisted from each other by 16.6°, 9.3°, and 0° respectively, with WSU-12(Cu) possessing an ideal paddlewheel node. In contrast, the WSU-20 structure features a more distorted cluster; here, the metal-metal line has angled 12° away from the normal of the metal coordination planes, and the individual polyhedra are twisted 8.7° from each other. It is not DMAc coordination that directly causes the distortion; the nodes of WSU-12(Cu) were also resolved with two capping DMAc molecules and still display an ideal geometry. Instead, it is the proximity of adjacent DMAc-capped nodes that sterically induces the distortion, since ideal paddlewheel geometry would have resulted in direct DMAc overlap. The emerging relationships between node capping, ligand geometry, and framework dimensionality in this series led us to speculate a mechanistic connection between the WSU-10 and -20 phases.

**FIGURE 8:**
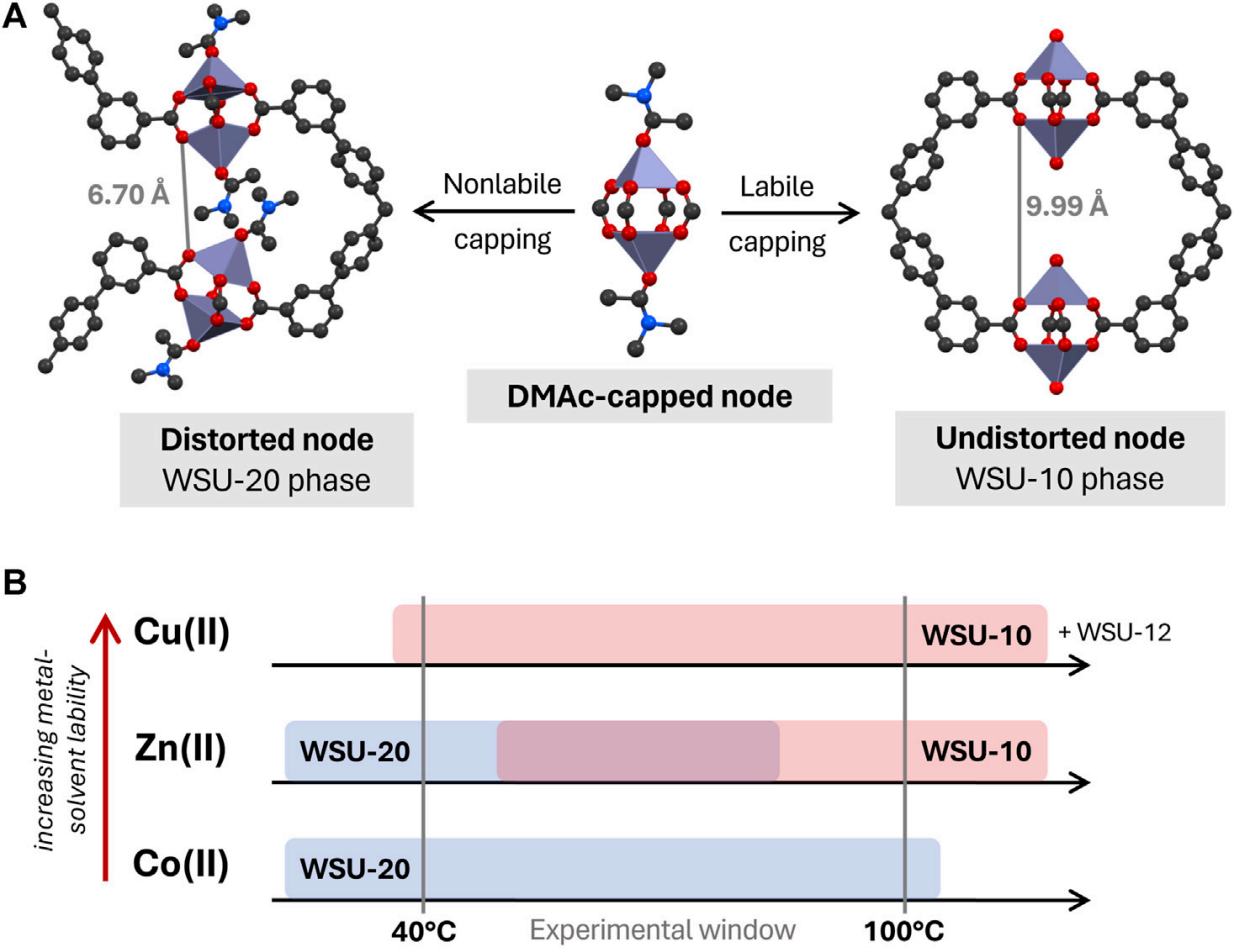
**(A)** Proposed relationship between WSU-10 and WSU-20 phases, based upon lability of the metal-DMAc capping interaction, and **(B)** diagram of observed phase occurrence by metal species. (Atom representations: metals are purple, carbon is black, oxygen is red, and nitrogen is blue; hydrogens omitted).

Consider a paddlewheel node that has formed in solution and is dynamically capped with solvent molecules (Figure 8). To form WSU-10 or -20, an *m*-ETTC ligand first equatorially chelates one cluster, and then must chelate a second cluster ‘below’ the first one in a way that axially aligns the two SBUs. However, the space between the axial tips of the clusters is not sufficient to allow two sets of solvent caps; the DMAc molecule of the top cluster would overlap directly with the DMAc cap of the lower cluster. Instead, there are two distinct routes for framework propagation: 1) retention of the solvent caps but distortion of the paddlewheel, allowing the two adjacent capped clusters to reside (as in WSU-20), or 2) loss of the bulky solvent caps, maintaining ideal geometry and spacing between all clusters (as in WSU-10). If route one occurs, the distorted clusters occupy positions that prevent direct bridging with a single *m-*ETTC molecule on the opposite side, as the 6.70 Å space is too small (Figure 8A, left). Instead, two different ligands are required to coordinate these nodes, forcing the framework to form the 3D, solvent-capped WSU-20 phase. However, if propagation occurs through route 2, then no distortion is necessary and the nodes are in ideal geometries and positions for a single *m-*ETTC ligand to coordinate the 9.99 Å space on the opposite side (Figure 8A, right), forming the pseudo-2D uncapped WSU-10 structure.

Thus, we hypothesize that the kinetic dependence of WSU-10/-20 formation is related to the lability of the metal-solvent interaction during crystallization. By this logic, a metal with a high affinity for solvent coordination and a low rate of solvent self-exchange (*i.e.*, low lability) would be more likely to form the capped WSU-20 structure, while a metal with lower solvent affinity and high rates of solvent exchange (*i.e.*, high lability) would be more likely to form the uncapped WSU-10 structure. One common measure of metal-solvent lability is the well-established rate constants of H_2_O exchange in metal aqua complexes (Lincoln, 2005). Although the precise rate magnitudes would not apply in nonaqueous systems such as this, lability in these aqua species is primarily dictated by factors specific to the metal ion, such as surface charge density and *d* orbital occupancy. Temperature also has an effect on lability and exchange kinetics, generally increasing rates of exchange with increasing temperatures.

In the WSU-10/-20 system, we observe that there is in fact a phase dependency on the identity of the metal as well as the temperature of the reaction, supporting this hypothesis (Figure 8B). Using Cu(II), the uncapped WSU-10 phase was obtained across all temperature ranges, and the capped WSU-20 phase was never obtained. This indicates that the Cu-DMAc interaction is labile, even at room temperature, and there is never a need for the node to distort in order to propagate a framework. Conversely, using Co(II), the WSU-20 phase is obtained even up to 100°C, and transition to the uncapped WSU-10 phase was not observed. This implies that the nature of the Co-DMAc interaction is quite strong (*i.e.*, nonlabile) and node distortion is preferred, even at high temperatures. Zn(II) was able to form both structures in a temperature-dependent manner. As expected, the uncapped WSU-10(Zn) phase was formed at higher temperatures (where metal-solvent lability is higher), while the distorted WSU-20(Zn) phase was achieved at lower temperatures, where the metal-solvent interaction is less labile. This broad trend of metal-solvent lability across these metal ions, increasing from Co(II) < Zn(II) < Cu(II), is reflected exactly in the H_2_O exchange rates of their metal aqua complexes (Lincoln, 2005). It is the combined influence of the metal ion and the reaction temperature that controls the rate of metal-solvent exchange on the paddlewheel SBU, which in turn has a profound impact on the MOF phase that is obtained. The effects of reaction temperature (Yuan et al., 2016a; 2016b) and solvent identity (Zhang B. et al., 2015; Seetharaj et al., 2019) on MOF syntheses have been reported in a number of examples before. These works mainly focus on the solubility and acidity of the solvent; here, the kinetics of the solvent interaction with the metal ion appear to play the largest role.

One might wonder how the WSU-12 structure fits in to this mechanism, since it is a constitutionally isomeric phase to WSU-10 and -20 as well. This MOF was only obtained with Cu and, despite the established lability of Cu(II), was resolved with DMAc molecules coordinated to the nodes. Here, we propose that since the WSU-12 structure allows typical paddlewheel geometry while capped, there is no energetic need for the node to follow one particular route. The metal-solvent capping interaction is not in fact related to the assembly of WSU-12, disqualifying this phase from the same dependence on lability that the WSU-10 and -20 phases experience. This kinetic independence suggests that both the WSU-12(Zn) and WSU-12(Co) phases may also be accessible if ligand conformation can be controlled. Unfortunately, the synthetic factors that encourage specific ligand orientations are far less direct than the kinetic control on node geometry demonstrated here, and the WSU-12 phase appears in this study to be less energetically favorable than alternatives with planar, symmetric *m-*ETTC conformations.

In summary, a series of coordination polymers (WSU-10, -11, −12, and −20) were synthesized based on Zn, Cu, and Co paddlewheel metal nodes and the TPE-based ligand *m-*H_4_ETTC, featuring a variety of ligand and node conformations that influence their dimensionalities. WSU-20 is a kinetic isomer of WSU-10, and a mechanistic relationship between the two phases has been presented that relies on the lability of the metal-solvent interaction during synthesis. This kinetic influence on phase distribution could lead to further understanding and control over the MOF construction process. Additionally, these new structures provide a better picture of the conformation that the lower-symmetry *m-*H_4_ETTC ligand can adopt with paddlewheel nodes to propagate extended frameworks. Continued synthetic explorations are being performed to further probe the kinetic landscape of the *m-*ETTC system and expand this isomeric series.

## Data Availability

The original contributions presented in the study are included in the article/Supplementary Material; further inquiries can be directed to the corresponding authors. The data presented in the study are deposited in the Cambridge Crystallographic Data Centre (CCDC) repository, accession numbers 2295734–2295739. These data can be obtained free of charge via www.ccdc.cam.ac.uk/data_request/cif, or by emailing data_request@ccdc.cam.ac.uk, or by contacting The Cambridge Crystallographic Data Centre, 12 Union Road, Cambridge CB2 1EZ, United Kingdom; fax: +44 1223 336033.
