# Supplementary material for: Probing ligand conformation and net dimensionality in a series of tetraphenylethene-based metal–organic frameworks
Source: Front Chem. 2024 Apr 25;12:1396123. doi: 10.3389/fchem.2024.1396123 (PMC11079141; doi:10.3389/fchem.2024.1396123)
Supplement: Supplementary file 1 [file DataSheet7.pdf]

Supplementary Material

Table S1. Crystallographic data of compounds.

Compound	**WSU-10(Cu)**	**WSU-11(Cu)**	**WSU-11(Zn)**	**WSU-12(Cu)**
CCDC No.	2295734	2295735	2295736	2295739
Empirical Formula	$Cu_2C_{54}H_{32}O_9$	$Cu_2C_{56}H_{38}O_9N$	$Zn_2C_{56}H_{34}O_9N$	$Cu_2C_{62}H_{50}N_2O_{10}$
Formula Weight	951.87	995.95	995.58	1110.12
Temperature (K)	273.15	298	273.15	149.99
Crystal System	tetragonal	tetragonal	tetragonal	monoclinic
Space Group	*P*4/*nnc*	*P*4/*nnc*	*P*4/*nnc*	*C*2/*c*
a (Å)	19.3354(19)	19.1840(9)	19.5604(11)	27.190(5)
b (Å)	19.3354(19)	19.1840(9)	19.5604(11)	29.265(6)
c (Å)	20.825(2)	22.0403(12)	21.8824(14)	20.527(4)
α (°)	90	90	90	90
β (°)	90	90	90	112.081(8)
γ (°)	90	90	90	90
V ($Å^3$)	7785.5(17)	8111.4(9)	8372.4(11)	15136(5)
Z	4	4	4	8
ρ_{calc} (g/cm^3)	0.812	0.816	0.790	0.974
F (000)	1944	2044	2036	4592
Wavelength (Å)	0.71073	0.71073	0.71073	0.71073
Reflections Collected	53440	75945	130752	83801
Independent Reflections	1060	3481	3593	12917
R_1 (residues)	0.0810	0.0523	0.0482	0.0751
wR_2 (residues)	0.2463	0.1902	0.1807	0.2492
GOF	1.115	0.952	1.090	1.049

Crystallographic data of compounds continued.

Compound	**WSU-20(Co)**	**WSU-20(Zn)**
CCDC no.	2295737	2295738
Empirical Formula	$Co_4C_{116}H_{80}N_3O_{20}$	$Zn_2C_{62}H_{50}N_2O_{10}$
Formula Weight	2071.55	1113.78
Temperature (K)	273.15	273.15
Crystal System	monoclinic	monoclinic
Space Group	*C2/c*	*C2/c*
a (Å)	33.738(4)	33.730(3)
b (Å)	28.170(3)	28.276(3)
c (Å)	20.559(2)	19.9896(18)
α (°)	90	90
β (°)	122.939(3)	122.708(3)
γ (°)	90	90
V (Å^3)	16398(3)	16042(3)
Z	4	8
ρ_{calc} (g/cm^3)	0.839	0.922
F (000)	4260	4608
Wavelength (Å)	0.71073	0.71073
Reflections Collected	86337	105959
Independent Reflections	13955	13655
R_1 (residues)	0.1022	0.0727
wR_2 (residues)	0.3041	0.2245
GOF	1.064	1.066

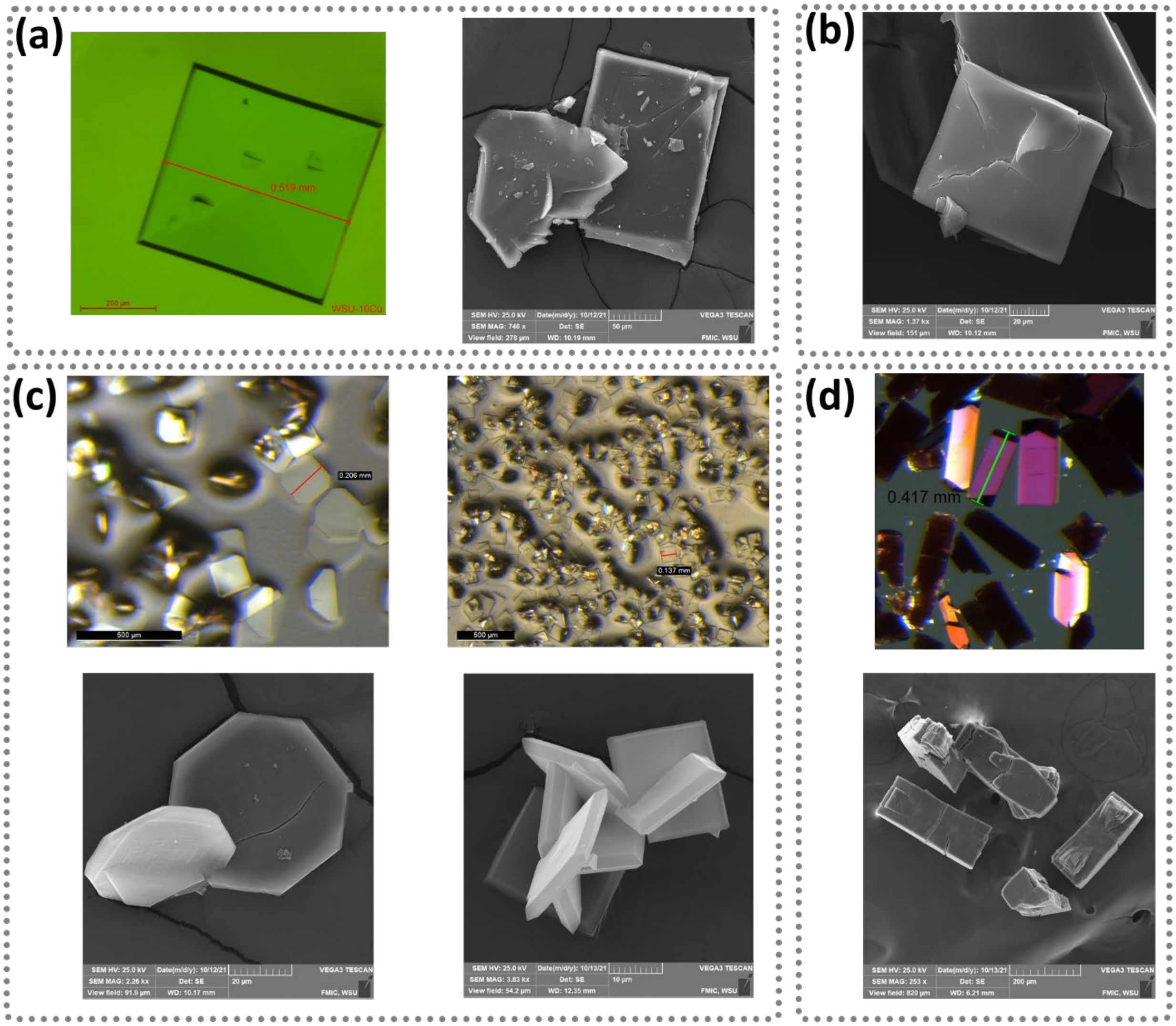

Figure S1: Light microscope and scanning electron microscopy (SEM) images of (A) WSU-10(Cu), (B) WSU-11(Cu), (C) WSU-10(Zn), and (D) WSU-20(Co).

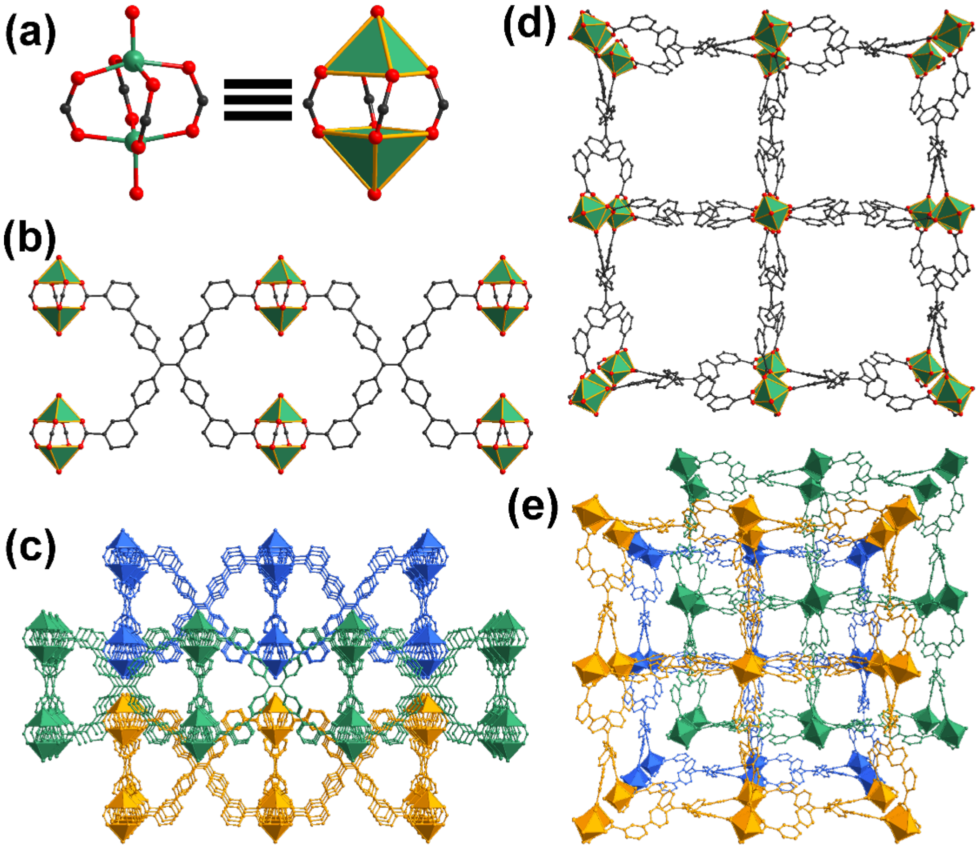

Figure S2: Illustration of the structure of WSU-10(Cu). (A) The paddlewheel Cu2 cluster in WSU-10(Cu); (B) the orientation of m-ETTC ligands in a single layer; (C) depiction of the stacking of three layers in WSU-10(Cu), as viewed along the b-axis; (D) depiction of a single layer, as viewed along the c-axis, and (E) depiction of the stacking of three layers in WSU-10(Cu), as viewed along the c-axis. (Atom representations: copper is green, carbon is black, oxygen is red, hydrogens omitted).

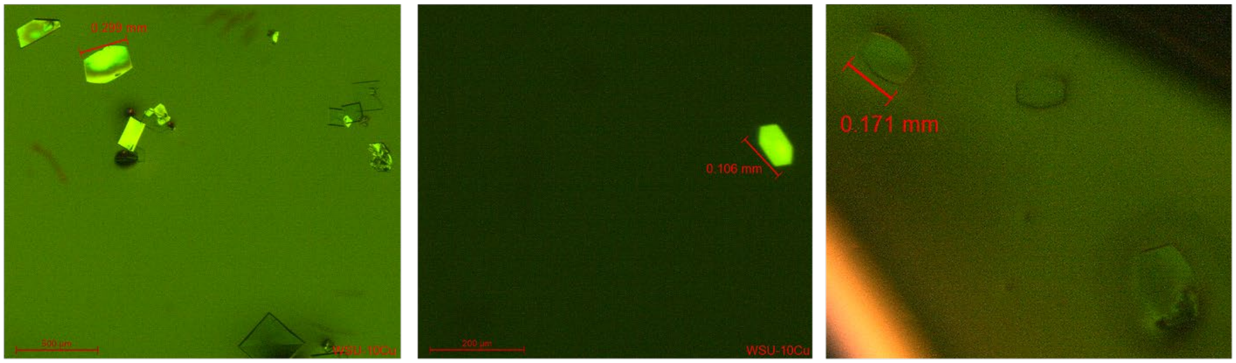

Figure S3: Light microscope images of the crystals of WSU-12(Cu), taken from vials of WSU-10(Cu).

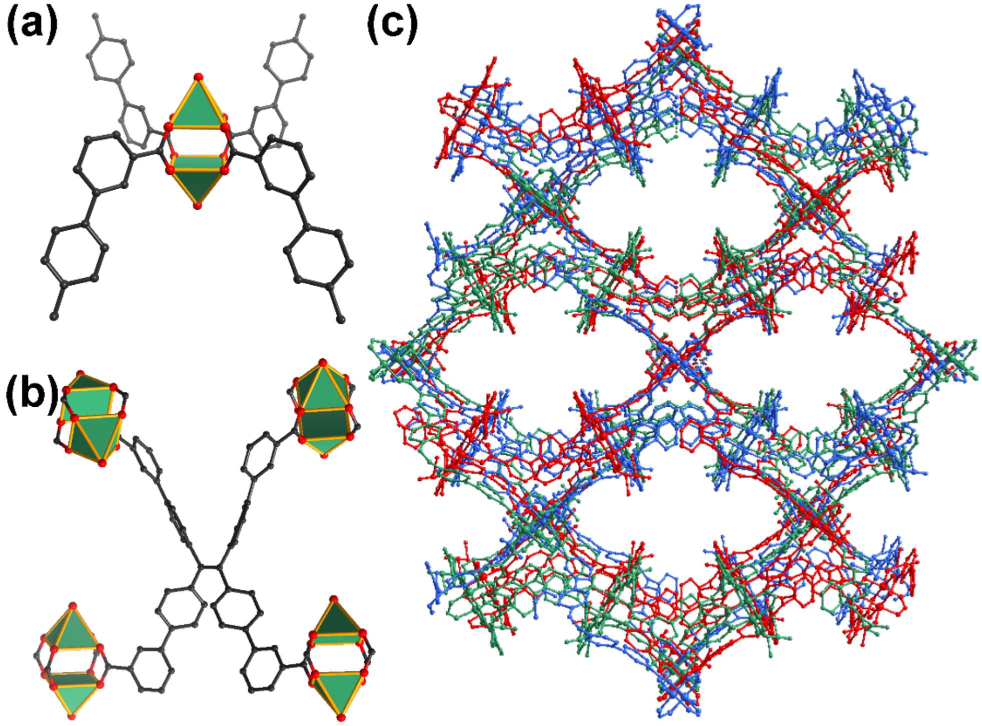

Figure S4. Illustration of the structure of WSU-12(Cu). (A) The DMAc-capped paddlewheel node, showing two ligands connected from below the plane and two from above; (B) view of one ligand bent out-of-plane, connected to four Cu_2 clusters; (C) depiction of the 3-fold interpenetrated structure, as viewed along the c-axis. (Atom representations: copper is green, carbon is black, oxygen is red, hydrogens omitted).

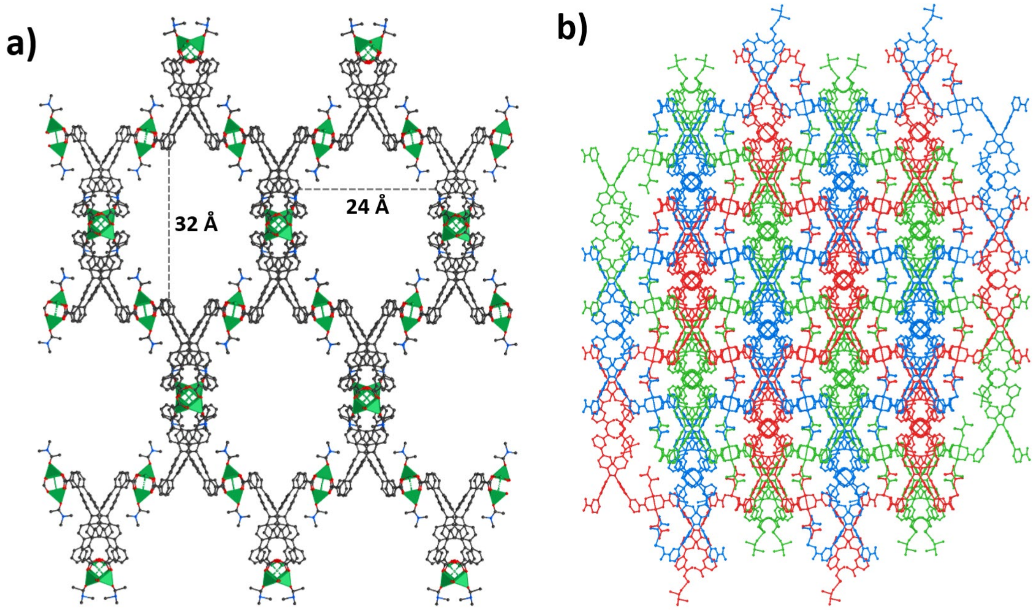

Figure S5: Additional structural images of WSU-12(Cu), showing (A) the largest channel of a single net and (B) the filling of this channel by three-fold interpenetration in the bulk material.

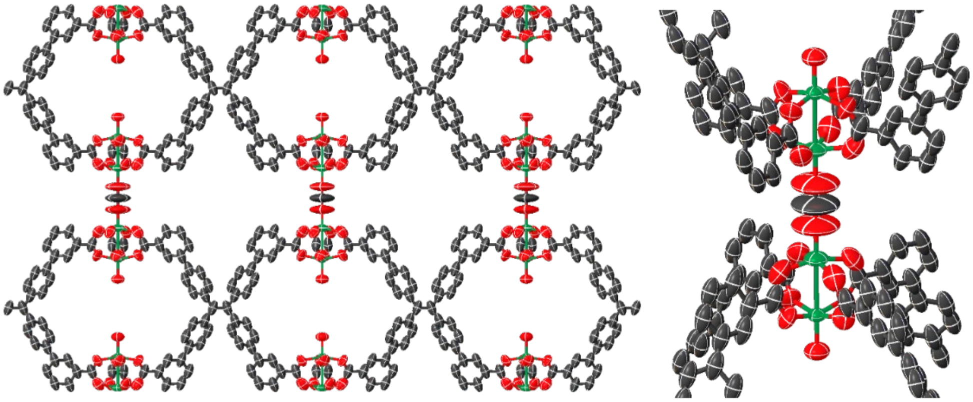

Figure S6: Experimentally determined structure of WSU-10(Cu) showing disordered CO_2 bridging adjacent layers. Thermal ellipsoids drawn at 50% probability.

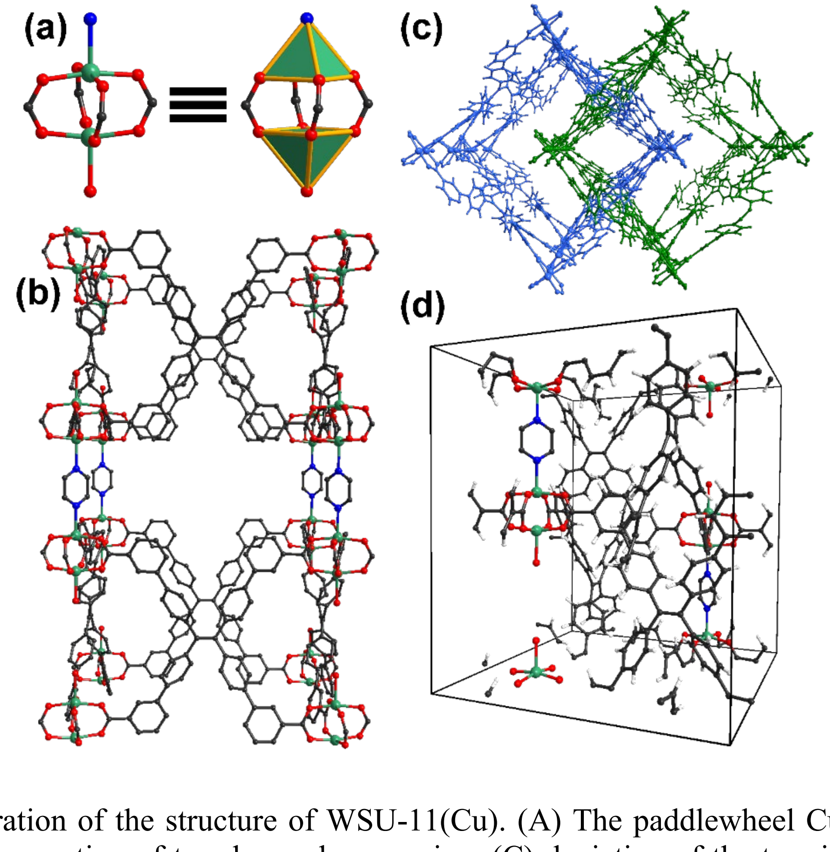

Figure S7. Illustration of the structure of WSU-11(Cu). (A) The paddlewheel Cu_2 cluster in WSU-10(Cu); (B) the connection of two layers by pyrazine; (C) depiction of the two independent nets of WSU-11(Cu), as viewed along the c-axis; (D) the unit cell of WSU-11(Cu) depicting the connecting of two pairs of layers. (Atom representations: copper is green, carbon is black, oxygen is red, nitrogen is blue, hydrogens omitted).

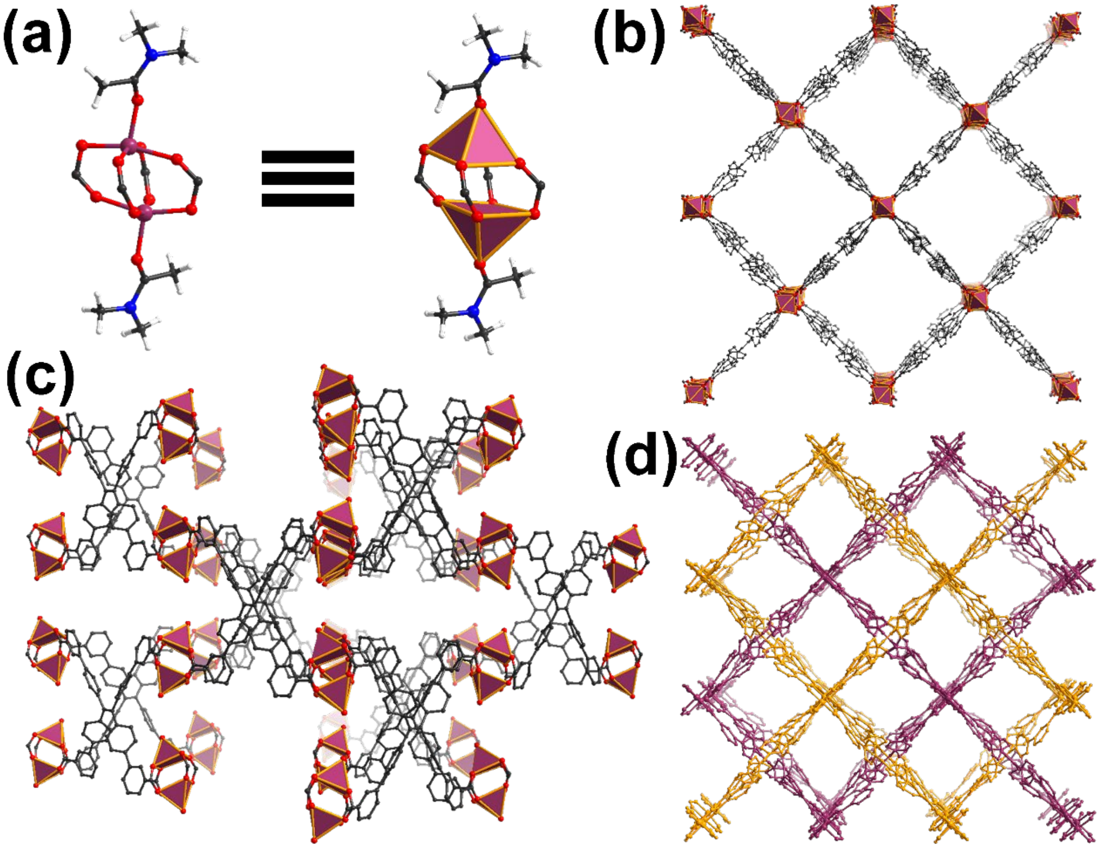

Figure S8. Illustration of the structure of WSU-20(Co). (A) The distorted paddlewheel Co_2 cluster in WSU-20(Co); (B) depiction of the channel pores of a single net of WSU-20(Co), as viewed along the c-axis; (C) depiction of WSU-20(Co) as viewed along the b-axis; (D) depiction of the 2-fold interpenetrated structure of WSU-20(Co), as viewed along the c-axis. (Atom representations: cobalt is pink, carbon is black, oxygen is red, nitrogen is blue, and hydrogen is white).

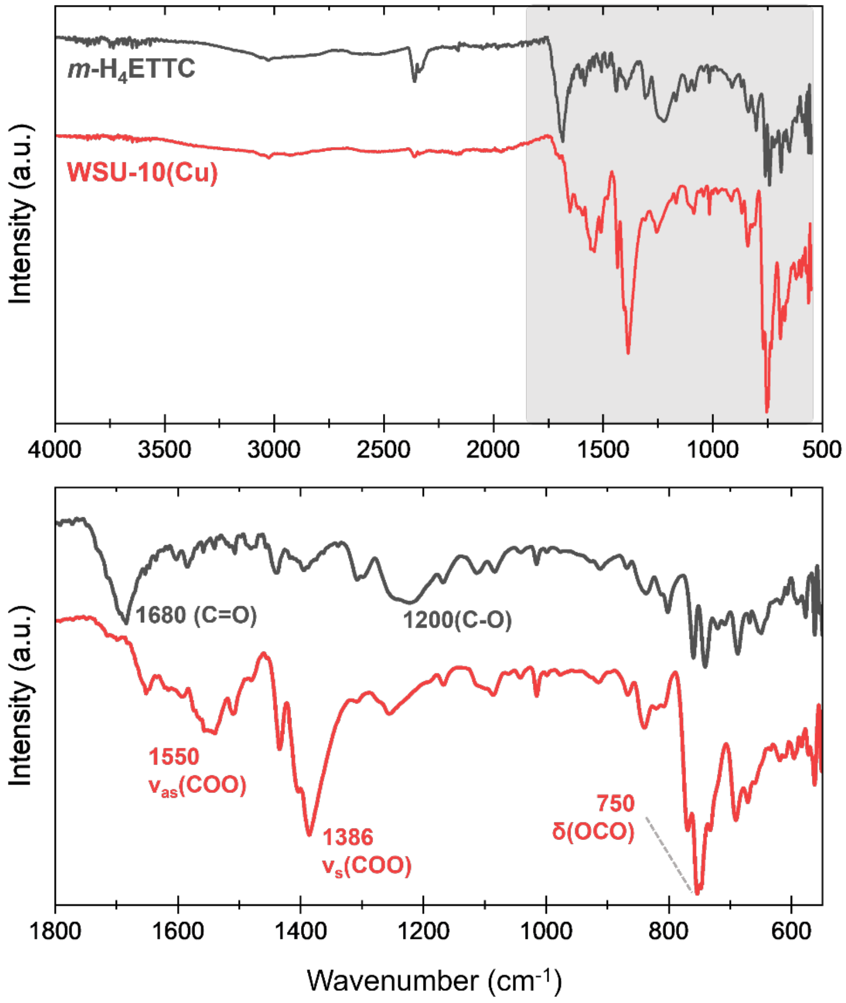

Figure S9: Solid-state FTIR spectra of the *m*-H_4ETTC linker (black) and WSU-10(Cu) (red). Top: full spectrum; bottom: 550 – 1800 cm^{-1} region.

In *m*-H_4ETTC, the distinct C=O and C-O stretches of the free carboxylate at 1680 and ~1200 cm^{-1} reflect their respective bond orders of 2 and 1. In WSU-10, the coordinated carboxylate demonstrates two ~1.5 order C--O bonds with median stretching energies (1550 and 1386 cm^{-1} for the asymmetric and symmetric stretches, respectively). The signal at ~750 cm^{-1} has contributions from various bending modes of the carboxylate (OCO) and phenyl rings (C-C-C and aromatic -H). [1,2]

[1] Gentile, S.F., Pannico, M., Causa, M., Mensitieri G., Di Palma, G., Scherillo, G., and Musto, P. *J. Mater. Chem. A,* 2020, **8**, 10796-10812

[2] Max, J.-J. and Chapados, C. *J. Phys. Chem. A,* 2002, 106, 27, 6452-6461

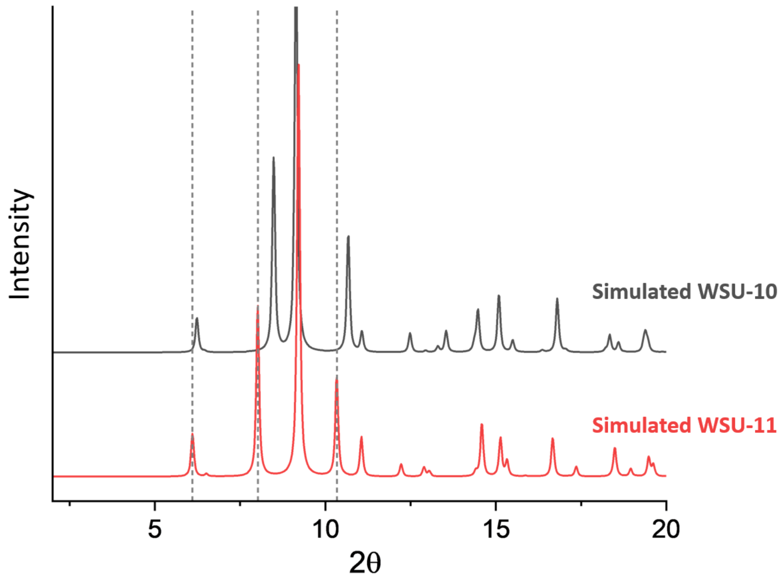

Figure S10: Comparison of the simulated powder XRD patterns of WSU-10 and WSU-11 below 20°. Dashed lines mark prominent diffraction features that have been noticeably shifted to lower angles in WSU-11 as a result of the increased interlayer spacing.

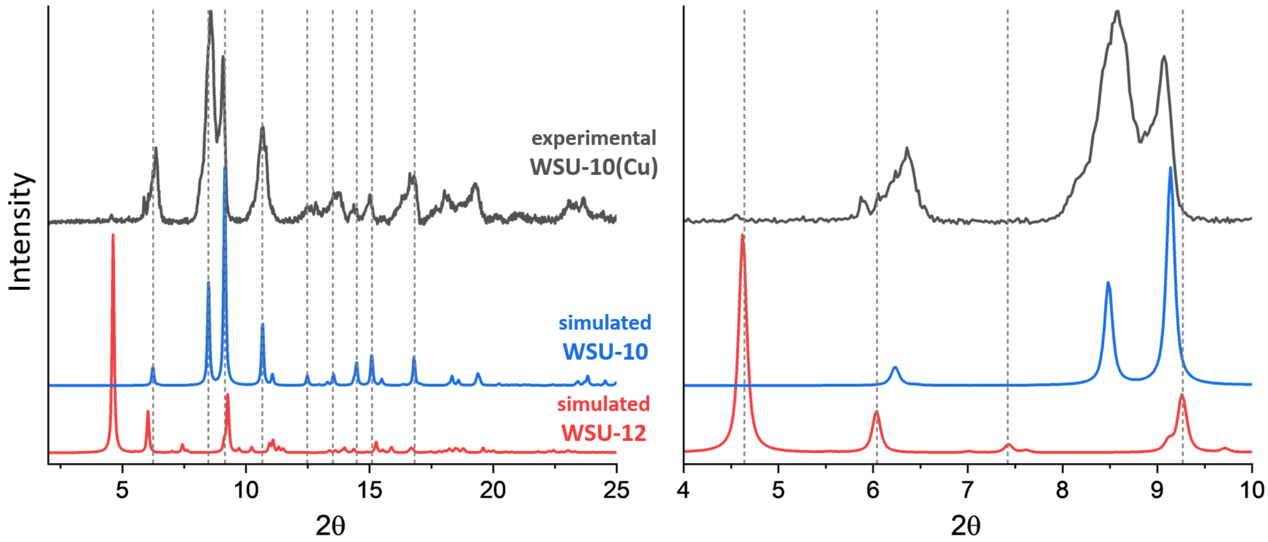

Figure S11: Experimental powder XRD pattern of synthesized WSU-10(Cu) sample compared to the simulated patterns for the WSU-10 and WSU-12 structures. Despite the identification of WSU-12(Cu) from vials of WSU-10(Cu), negligible diffraction contribution from the WSU-12 structure is observed in bulk samples of WSU-10(Cu), confirming the largely phase-pure nature of these reactions.

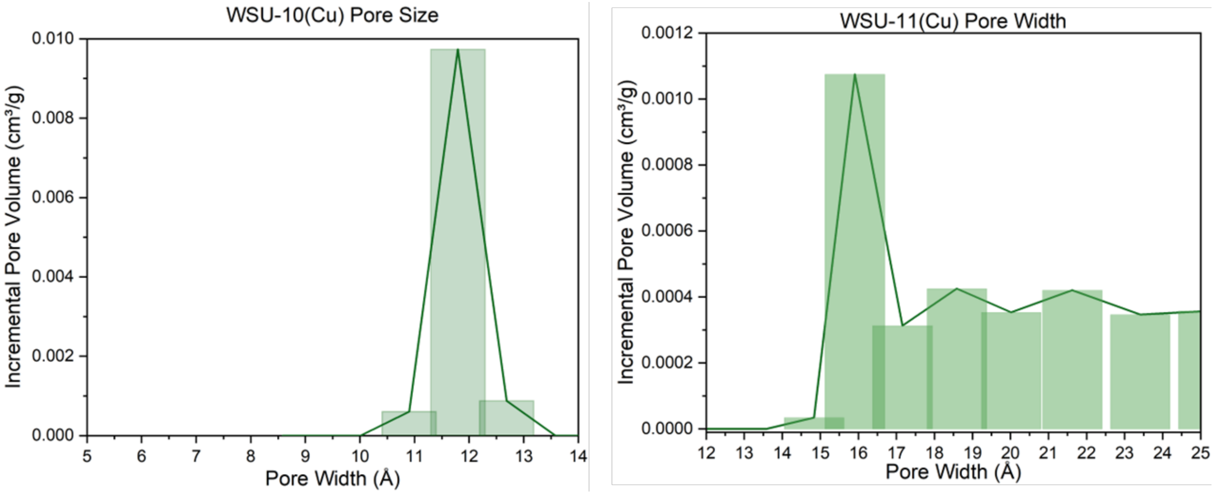

Figure S12: Experimental pore size distributions from N_2 adsorption analysis of WSU-10(Cu) (left) and WSU-11(Cu) (right).

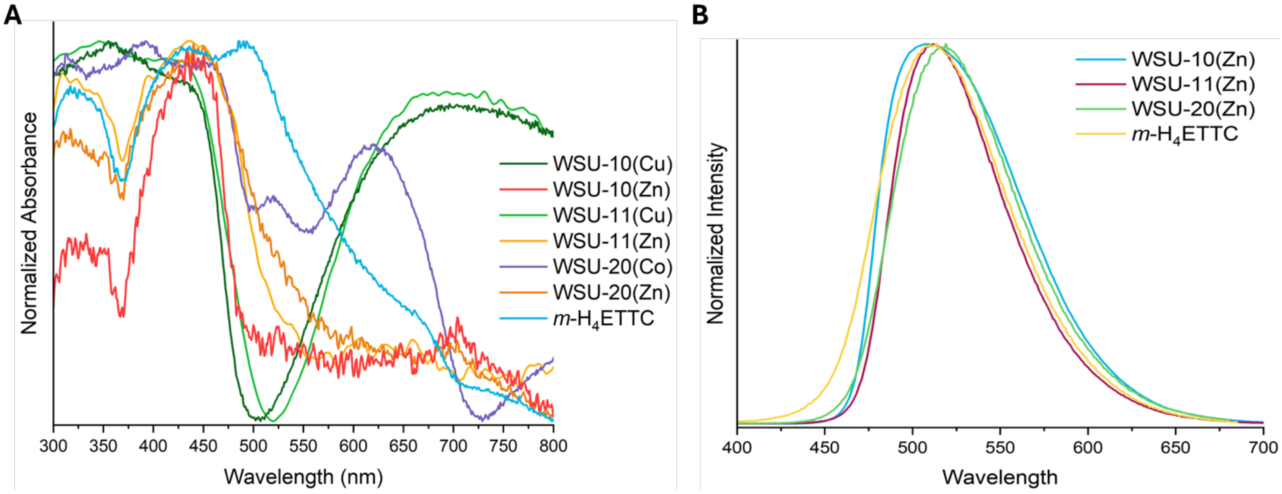

Figure S13: (A) Diffuse reflectance UV-Vis absorbance spectra of all WSU-10, -11, and -20 MOFs and the free *m*-H_4ETTC linker. The optically innocent Zn species predominantly show features of the pure linker, while those of optically active metals show those characteristic features as well. (B) Solid-state fluorescence emission spectra (λ_{ex} = 365 nm) of all Zn-based compounds compared to the free *m*-H_4ETTC linker.
