# Supplementary material for: Probing ligand conformation and net dimensionality in a series of tetraphenylethene-based metal–organic frameworks
Source: Front Chem. 2024 Apr 25;12:1396123. doi: 10.3389/fchem.2024.1396123 (PMC11079141; doi:10.3389/fchem.2024.1396123)

checkCIF/PLATON report

Structure factors have been supplied for datablock(s) mjh_xrd_8_0m

THIS REPORT IS FOR GUIDANCE ONLY. IF USED AS PART OF A REVIEW PROCEDURE FOR PUBLICATION, IT SHOULD NOT REPLACE THE EXPERTISE OF AN EXPERIENCED CRYSTALLOGRAPHIC REFEREE.

No syntax errors found. CIF dictionary Interpreting this report

Datablock: mjh_xrd_8_0m

Bond precision: C-C = 0.0049 A Wavelength=0.71073

Cell:	a=19.1840(9)	b=19.1840(9)	c=22.0403(12)
	alpha=90	beta=90	gamma=90
Temperature:	298 K		

	Calculated	Reported
Volume	8111.4(9)	8111.4(9)
Space group	P 4/n n c	P 4/n n c
Hall group	-P 4a 2bc	-P 4a 2bc
Moiety formula	C56 H38 Cu2 N O9 [+ solvent]	0.5(C112 H76 Cu4 N2 O18)
Sum formula	C56 H38 Cu2 N O9 [+ solvent]	C56 H38 Cu2 N O9
Mr	995.97	995.95
Dx,g cm-3	0.816	0.816
Z	4	4
Mu (mm-1)	0.559	0.559
F000	2044.0	2044.0
F000’	2047.39	
h,k,lmax	22,22,26	22,21,24
Nref	3510	3481
Tmin,Tmax	0.894,0.959	0.630,0.745
Tmin’	0.894	

Correction method= # Reported T Limits: Tmin=0.630 Tmax=0.745
AbsCorr = MULTI-SCAN

Data completeness= 0.992 Theta(max)= 24.788

R(reflections)= 0.0523(1811) wR2(reflections)= 0.1902(3481)

S = 0.952 Npar= 168

```
The following ALERTS were generated. Each ALERT has the format
       test-name_ALERT_alert-type_alert-level.
Click on the hyperlinks for more details of the test.
```

Alert level B

```
PLAT031_ALERT_4_B Refined Extinction Parameter Within Range ......      2.250 Sigma
PLAT420_ALERT_2_B D-H Without Acceptor       O3        --H3A      .     Please Check
PLAT420_ALERT_2_B D-H Without Acceptor       O3        --H3B      .     Please Check
PLAT430_ALERT_2_B Short Inter D...A Contact  N1        ..N1       .       2.70 Ang.
                                                  y,x,1/2-z  =      7_555 Check
```

Alert level C

```
THETM01_ALERT_3_C  The value of sine(theta_max)/wavelength is less than 0.590
            Calculated sin(theta_max)/wavelength =    0.5899
PLAT222_ALERT_3_C NonSolvent Resd 1  H   Uiso(max)/Uiso(min) Range        5.5 Ratio
PLAT232_ALERT_2_C Hirshfeld Test Diff (M-X)  Cu1       --O3       .        8.0 s.u.
PLAT234_ALERT_4_C Large Hirshfeld Difference N1        --C1A      .       0.16 Ang.
PLAT242_ALERT_2_C Low    MainMol Ueq as Compared to Neighbors of        Cu1 Check
PLAT906_ALERT_3_C Large K Value in the Analysis of Variance ......      5.970 Check
PLAT911_ALERT_3_C Missing FCF Refl Between Thmin & STh/L=    0.590         12 Report
PLAT918_ALERT_3_C Reflection(s) with I(obs) much Smaller I(calc) .          1 Check
PLAT934_ALERT_3_C Number of (Iobs-Icalc)/Sigma(W) > 10 Outliers ..          1 Check
PLAT976_ALERT_2_C Check Calcd Resid. Dens.  0.84A   From O3             -0.46 eA-3
PLAT978_ALERT_2_C Number C-C Bonds with Positive Residual Density.          0 Info
```

Alert level G

```
PLAT002_ALERT_2_G Number of Distance or Angle Restraints on AtSite          4 Note
PLAT004_ALERT_5_G Polymeric Structure Found with Maximum Dimension          3 Info
PLAT007_ALERT_5_G Number of Unrefined Donor-H Atoms ..............          2 Report
PLAT042_ALERT_1_G Calc. and Reported MoietyFormula Strings  Differ     Please Check
PLAT072_ALERT_2_G SHELXL First  Parameter in WGHT  Unusually Large       0.12 Report
PLAT169_ALERT_4_G The CIF-Embedded .res File Contains AFIX 1 Recds          2 Report
PLAT171_ALERT_4_G The CIF-Embedded .res File Contains EADP Records          1 Report
PLAT172_ALERT_4_G The CIF-Embedded .res File Contains DFIX Records          3 Report
PLAT300_ALERT_4_G Atom Site Occupancy of H3A        Constrained at        0.5 Check
PLAT300_ALERT_4_G Atom Site Occupancy of H3B        Constrained at        0.5 Check
PLAT300_ALERT_4_G Atom Site Occupancy of H1A        Constrained at       0.25 Check
PLAT300_ALERT_4_G Atom Site Occupancy of H1B        Constrained at       0.25 Check
PLAT301_ALERT_3_G Main Residue  Disorder ..............(Resd  1  )         3% Note
PLAT432_ALERT_2_G Short Inter X...Y Contact  N1        ..C1A             2.30 Ang.
                                              x,3/2-y,1/2-z  =      6_565 Check
PLAT432_ALERT_2_G Short Inter X...Y Contact  N1        ..C1B             2.33 Ang.
                                              x,3/2-y,1/2-z  =      6_565 Check
PLAT606_ALERT_4_G VERY LARGE Solvent Accessible VOID(S) in Structure        ! Info
PLAT860_ALERT_3_G Number of Least-Squares Restraints .............          3 Note
PLAT910_ALERT_3_G Missing # of FCF Reflection(s) Below Theta(Min).          4 Note
PLAT913_ALERT_3_G Missing # of Very Strong Reflections in FCF ....          2 Note
PLAT952_ALERT_5_G Calculated (ThMax) and CIF-Reported Lmax Differ           2 Units
PLAT958_ALERT_1_G Calculated (ThMax) and Actual (FCF) Lmax Differ           2 Units
PLAT961_ALERT_5_G Dataset Contains no Negative Intensities .......     Please Check
PLAT992_ALERT_5_G Repd & Actual _reflns_number_gt Values Differ by          1 Check
```

```
   0 ALERT level A = Most likely a serious problem - resolve or explain
   4 ALERT level B = A potentially serious problem, consider carefully
  11 ALERT level C = Check. Ensure it is not caused by an omission or oversight
  23 ALERT level G = General information/check it is not something unexpected
```

```
 2 ALERT type 1 CIF construction/syntax error, inconsistent or missing data
11 ALERT type 2 Indicator that the structure model may be wrong or deficient
10 ALERT type 3 Indicator that the structure quality may be low
10 ALERT type 4 Improvement, methodology, query or suggestion
 5 ALERT type 5 Informative message, check
```

It is advisable to attempt to resolve as many as possible of the alerts in all categories. Often the minor alerts point to easily fixed oversights, errors and omissions in your CIF or refinement strategy, so attention to these fine details can be worthwhile. In order to resolve some of the more serious problems it may be necessary to carry out additional measurements or structure refinements. However, the purpose of your study may justify the reported deviations and the more serious of these should normally be commented upon in the discussion or experimental section of a paper or in the "special_details" fields of the CIF. checkCIF was carefully designed to identify outliers and unusual parameters, but every test has its limitations and alerts that are not important in a particular case may appear. Conversely, the absence of alerts does not guarantee there are no aspects of the results needing attention. It is up to the individual to critically assess their own results and, if necessary, seek expert advice.

Publication of your CIF in IUCr journals

A basic structural check has been run on your CIF. These basic checks will be run on all CIFs submitted for publication in IUCr journals (*Acta Crystallographica*, *Journal of Applied Crystallography*, *Journal of Synchrotron Radiation*); however, if you intend to submit to *Acta Crystallographica Section C* or *E* or *IUCrData*, you should make sure that full publication checks are run on the final version of your CIF prior to submission.

Publication of your CIF in other journals

Please refer to the *Notes for Authors* of the relevant journal for any special instructions relating to CIF submission.

PLATON version of 22/12/2019; check.def file version of 13/12/2019

Datablock mjh_xrd_8_0m - ellipsoid plot

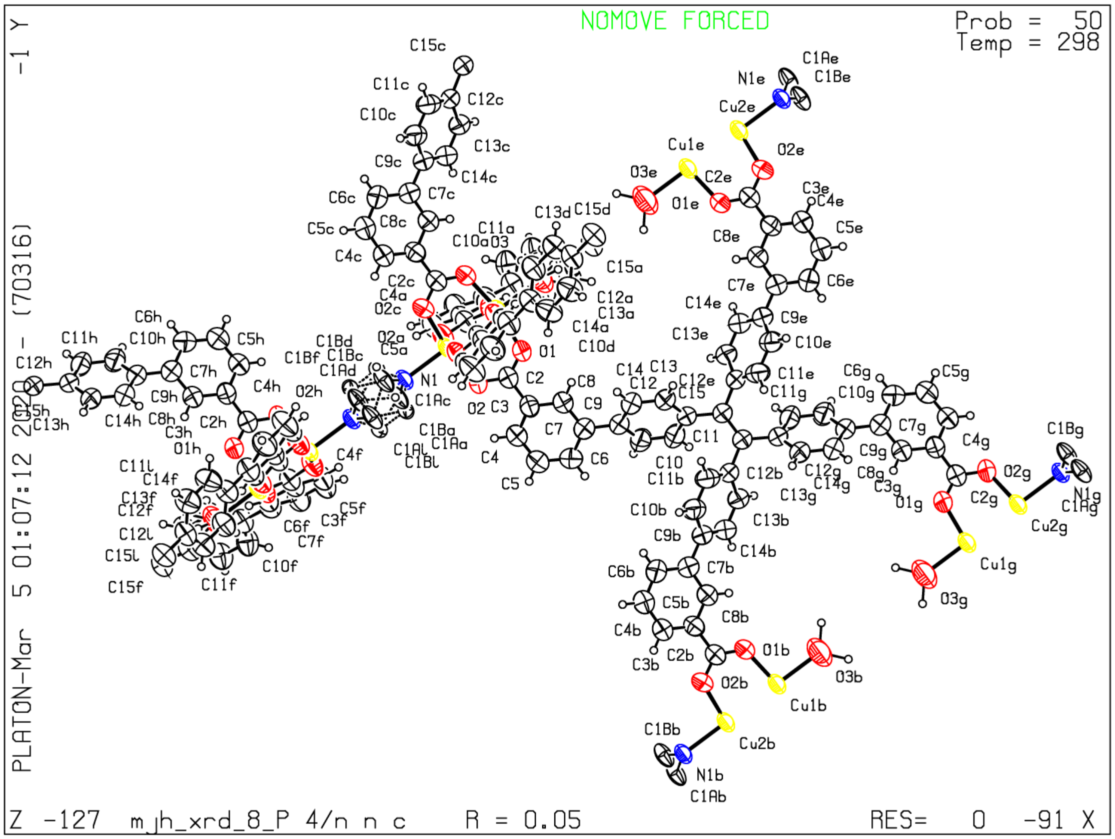

Supplement: Supplementary file 2 [file DataSheet2.PDF]
