# Supplementary material for: Probing ligand conformation and net dimensionality in a series of tetraphenylethene-based metal–organic frameworks
Source: Front Chem. 2024 Apr 25;12:1396123. doi: 10.3389/fchem.2024.1396123 (PMC11079141; doi:10.3389/fchem.2024.1396123)

checkCIF/PLATON report

Structure factors have been supplied for datablock(s) 10cu_0m

THIS REPORT IS FOR GUIDANCE ONLY. IF USED AS PART OF A REVIEW PROCEDURE FOR PUBLICATION, IT SHOULD NOT REPLACE THE EXPERTISE OF AN EXPERIENCED CRYSTALLOGRAPHIC REFEREE.

No syntax errors found. CIF dictionary Interpreting this report

Datablock: 10cu_0m

Bond precision:	C-C = 0.0079 A		Wavelength=0.71073
Cell:	a=27.190(5)	b=29.265(6)	c=20.527(4)
	alpha=90	beta=112.081(8)	gamma=90
Temperature:	150 K		

	Calculated	Reported
Volume	15136(5)	15136(5)
Space group	C 2/c	C 1 2/c 1
Hall group	-C 2yc	-C 2yc
Moiety formula	C62 H50 Cu2 N2 O10 [+ solvent]	C62 H50 Cu2 N2 O10
Sum formula	C62 H50 Cu2 N2 O10 [+ solvent]	C62 H50 Cu2 N2 O10
Mr	1110.14	1110.12
Dx,g cm-3	0.974	0.974
Z	8	8
Mu (mm-1)	0.606	0.606
F000	4592.0	4592.0
F000'	4598.98	
h,k,lmax	32,34,24	32,34,24
Nref	13003	12917
Tmin,Tmax	0.702,0.941	0.627,0.745
Tmin'	0.648	

Correction method= # Reported T Limits: Tmin=0.627 Tmax=0.745
AbsCorr = MULTI-SCAN

Data completeness= 0.993 Theta(max)= 24.778

R(reflections)= 0.0751(9234) wR2(reflections)= 0.2492(12917)

S = 1.049 Npar= 598

The following ALERTS were generated. Each ALERT has the format
test-name_ALERT_alert-type_alert-level.
Click on the hyperlinks for more details of the test.

Alert level B

```
PLAT934_ALERT_3_B Number of (Iobs-Icalc)/Sigma(W) > 10 Outliers ..          2 Check
```

Alert level C

```
THETM01_ALERT_3_C  The value of sine(theta_max)/wavelength is less than 0.590
            Calculated sin(theta_max)/wavelength =    0.5897
PLAT218_ALERT_3_C Constrained U(ij) Components(s) for N1S        .           6 Check
PLAT218_ALERT_3_C Constrained U(ij) Components(s) for C1S        .           6 Check
PLAT218_ALERT_3_C Constrained U(ij) Components(s) for C2S        .           6 Check
PLAT218_ALERT_3_C Constrained U(ij) Components(s) for C3S        .           6 Check
PLAT218_ALERT_3_C Constrained U(ij) Components(s) for C4S        .           6 Check
PLAT220_ALERT_2_C NonSolvent   Resd 1  C   Ueq(max)/Ueq(min) Range        3.6 Ratio
PLAT222_ALERT_3_C NonSolvent Resd 1  H   Uiso(max)/Uiso(min) Range        4.3 Ratio
PLAT241_ALERT_2_C High   'MainMol' Ueq as Compared to Neighbors of        O2S Check
PLAT242_ALERT_2_C Low    'MainMol' Ueq as Compared to Neighbors of        Cu1 Check
PLAT242_ALERT_2_C Low    'MainMol' Ueq as Compared to Neighbors of        Cu2 Check
PLAT242_ALERT_2_C Low    'MainMol' Ueq as Compared to Neighbors of        N1S Check
PLAT341_ALERT_3_C Low Bond Precision on  C-C Bonds ..............    0.00789 Ang.
PLAT412_ALERT_2_C Short Intra XH3 .. XHn     H2SA     ..H4SB     .       1.81 Ang.
                                                        x,y,z  =      1_555 Check
PLAT741_ALERT_1_C Bond    Calc   1.2453(3), Rep     1.24550 ......    Missing s.u.
              N1S      -C1S             1_555   1_555 ........     #   29 Check
PLAT741_ALERT_1_C Bond    Calc   1.5038(3), Rep     1.50460 ......    Missing s.u.
              N1S      -C3S             1_555   1_555 ........     #   30 Check
PLAT741_ALERT_1_C Bond    Calc   1.4429(3), Rep     1.44520 ......    Missing s.u.
              N1S      -C4S             1_555   1_555 ........     #   31 Check
PLAT741_ALERT_1_C Bond    Calc   1.5258(3), Rep     1.52510 ......    Missing s.u.
              C1S      -C2S             1_555   1_555 ........     #   37 Check
PLAT742_ALERT_1_C Angle   Calc   115.26(1), Rep      115.40 ......    Missing s.u.
              C1S     -N1S     -C3S        1_555  1_555  1_555     #   41 Check
PLAT742_ALERT_1_C Angle   Calc   127.55(1), Rep      127.50 ......    Missing s.u.
              C1S     -N1S     -C4S        1_555  1_555  1_555     #   42 Check
PLAT742_ALERT_1_C Angle   Calc   115.02(1), Rep      115.00 ......    Missing s.u.
              C4S     -N1S     -C3S        1_555  1_555  1_555     #   43 Check
PLAT742_ALERT_1_C Angle   Calc   110.64(1), Rep      110.60 ......    Missing s.u.
              N1S     -C1S     -C2S        1_555  1_555  1_555     #   54 Check
PLAT743_ALERT_1_C Torsion Calc  -179.72(1), Rep     -179.70 ......    Missing s.u.
              C3S -N1S -C1S -C2S   1_555  1_555  1_555  1_555      #   32 Check
PLAT743_ALERT_1_C Torsion Calc   -17.43(1), Rep      -17.40 ......    Missing s.u.
              C4S -N1S -C1S -C2S   1_555  1_555  1_555  1_555      #   36 Check
PLAT767_ALERT_4_C INS Embedded LIST 6 Instruction Should be LIST 4     Please Check
PLAT905_ALERT_3_C Negative K value in the Analysis of Variance ...     -0.748 Report
PLAT910_ALERT_3_C Missing # of FCF Reflection(s) Below Theta(Min).          8 Note
PLAT911_ALERT_3_C Missing FCF Refl Between Thmin & STh/L=    0.590         75 Report
PLAT971_ALERT_2_C Check Calcd Resid. Dens.  1.08Ang From C3S             1.86 eA-3
```

```
PLAT972_ALERT_2_C Check Calcd Resid. Dens.  0.23Ang From C4S           -1.60 eA-3
PLAT977_ALERT_2_C Check Negative Difference Density on H4SC      .      -0.40 eA-3
```

Alert level G

```
PLAT004_ALERT_5_G Polymeric Structure Found with Maximum Dimension          3 Info
PLAT063_ALERT_4_G Crystal Size Possibly too Large for Beam Size ..       0.70 mm
PLAT072_ALERT_2_G SHELXL First  Parameter in WGHT  Unusually Large       0.15 Report
PLAT083_ALERT_2_G SHELXL Second Parameter in WGHT  Unusually Large      37.77 Why ?
PLAT169_ALERT_4_G The CIF-Embedded .res File Contains AFIX 1 Recds         29 Report
PLAT171_ALERT_4_G The CIF-Embedded .res File Contains EADP Records          3 Report
PLAT300_ALERT_4_G Atom Site Occupancy of C6SA       Constrained at        0.5 Check
PLAT300_ALERT_4_G Atom Site Occupancy of C6SB       Constrained at        0.5 Check
PLAT300_ALERT_4_G Atom Site Occupancy of C8SA       Constrained at        0.5 Check
PLAT300_ALERT_4_G Atom Site Occupancy of C8SB       Constrained at        0.5 Check
PLAT300_ALERT_4_G Atom Site Occupancy of H6SA       Constrained at        0.5 Check
PLAT300_ALERT_4_G Atom Site Occupancy of H6SB       Constrained at        0.5 Check
PLAT300_ALERT_4_G Atom Site Occupancy of H6SC       Constrained at        0.5 Check
PLAT300_ALERT_4_G Atom Site Occupancy of H6SD       Constrained at        0.5 Check
PLAT300_ALERT_4_G Atom Site Occupancy of H6SE       Constrained at        0.5 Check
PLAT300_ALERT_4_G Atom Site Occupancy of H6SF       Constrained at        0.5 Check
PLAT300_ALERT_4_G Atom Site Occupancy of H8SA       Constrained at        0.5 Check
PLAT300_ALERT_4_G Atom Site Occupancy of H8SB       Constrained at        0.5 Check
PLAT300_ALERT_4_G Atom Site Occupancy of H8SC       Constrained at        0.5 Check
PLAT300_ALERT_4_G Atom Site Occupancy of H8SD       Constrained at        0.5 Check
PLAT300_ALERT_4_G Atom Site Occupancy of H8SE       Constrained at        0.5 Check
PLAT300_ALERT_4_G Atom Site Occupancy of H8SF       Constrained at        0.5 Check
PLAT301_ALERT_3_G Main Residue  Disorder .............(Resd  1  )          3% Note
PLAT412_ALERT_2_G Short Intra XH3 .. XHn     H7SB     ..H8SF     .       1.97 Ang.
                                                    x,y,z  =      1_555 Check
PLAT606_ALERT_4_G Solvent Accessible VOID(S) in Structure .......           ! Info
PLAT720_ALERT_4_G Number of Unusual/Non-Standard Labels .........          28 Note
PLAT794_ALERT_5_G Tentative Bond Valency for Cu1       (II)      .       2.16 Info
PLAT868_ALERT_4_G ALERTS Due to the Use of _smtbx_masks Suppressed          ! Info
PLAT909_ALERT_3_G Percentage of I>2sig(I) Data at Theta(Max) Still        42% Note
PLAT913_ALERT_3_G Missing # of Very Strong Reflections in FCF ....          1 Note
PLAT933_ALERT_2_G Number of HKL-OMIT Records in Embedded .res File         10 Note
PLAT953_ALERT_1_G Reported (CIF) and Actual (FCF) Hmax Differ by .          1 Units
PLAT978_ALERT_2_G Number C-C Bonds with Positive Residual Density.          0 Info
```

---

0 **ALERT level A** = Most likely a serious problem - resolve or explain
1 **ALERT level B** = A potentially serious problem, consider carefully
31 **ALERT level C** = Check. Ensure it is not caused by an omission or oversight
33 **ALERT level G** = General information/check it is not something unexpected

11 ALERT type 1 CIF construction/syntax error, inconsistent or missing data
14 ALERT type 2 Indicator that the structure model may be wrong or deficient
15 ALERT type 3 Indicator that the structure quality may be low
23 ALERT type 4 Improvement, methodology, query or suggestion
2 ALERT type 5 Informative message, check

It is advisable to attempt to resolve as many as possible of the alerts in all categories. Often the minor alerts point to easily fixed oversights, errors and omissions in your CIF or refinement strategy, so attention to these fine details can be worthwhile. In order to resolve some of the more serious problems it may be necessary to carry out additional measurements or structure refinements. However, the purpose of your study may justify the reported deviations and the more serious of these should normally be commented upon in the discussion or experimental section of a paper or in the "special_details" fields of the CIF. checkCIF was carefully designed to identify outliers and unusual parameters, but every test has its limitations and alerts that are not important in a particular case may appear. Conversely, the absence of alerts does not guarantee there are no aspects of the results needing attention. It is up to the individual to critically assess their own results and, if necessary, seek expert advice.

Publication of your CIF in IUCr journals

A basic structural check has been run on your CIF. These basic checks will be run on all CIFs submitted for publication in IUCr journals (*Acta Crystallographica*, *Journal of Applied Crystallography*, *Journal of Synchrotron Radiation*); however, if you intend to submit to *Acta Crystallographica Section C* or *E* or *IUCrData*, you should make sure that full publication checks are run on the final version of your CIF prior to submission.

Publication of your CIF in other journals

Please refer to the *Notes for Authors* of the relevant journal for any special instructions relating to CIF submission.

PLATON version of 06/07/2023; check.def file version of 30/06/2023

Datablock 10cu_0m - ellipsoid plot

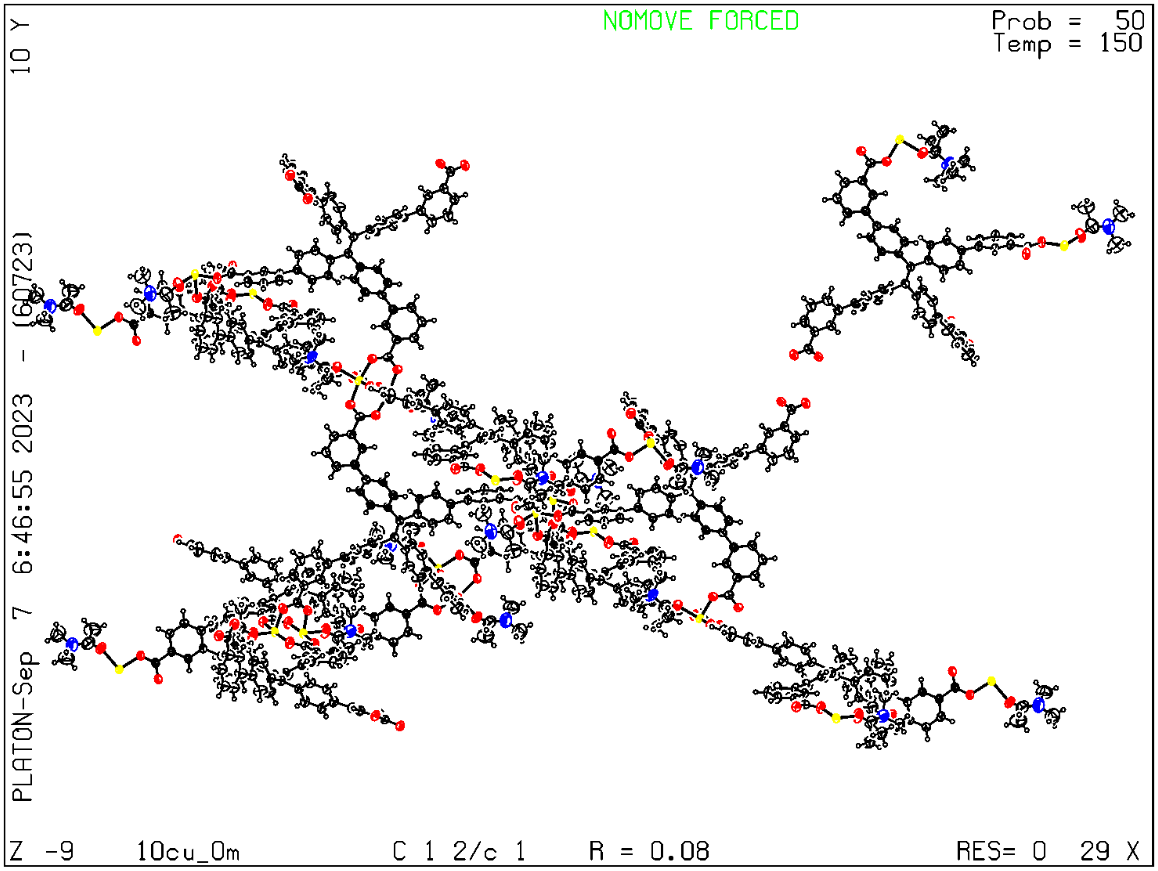

Supplement: Supplementary file 3 [file DataSheet4.PDF]
