# Supplementary material for: Probing ligand conformation and net dimensionality in a series of tetraphenylethene-based metal–organic frameworks
Source: Front Chem. 2024 Apr 25;12:1396123. doi: 10.3389/fchem.2024.1396123 (PMC11079141; doi:10.3389/fchem.2024.1396123)

checkCIF/PLATON report

Structure factors have been supplied for datablock(s) mjh_co_mettc_0m

THIS REPORT IS FOR GUIDANCE ONLY. IF USED AS PART OF A REVIEW PROCEDURE FOR PUBLICATION, IT SHOULD NOT REPLACE THE EXPERTISE OF AN EXPERIENCED CRYSTALLOGRAPHIC REFEREE.

No syntax errors found. CIF dictionary Interpreting this report

Datablock: mjh_co_mettc_0m

Bond precision:	C-C = 0.0115 A		Wavelength=0.71073
Cell:	a=33.738(4)	b=28.170(3)	c=20.559(2)
	alpha=90	beta=122.939(3)	gamma=90
Temperature:	273 K		

	Calculated	Reported
Volume	16398(3)	16398(3)
Space group	C 2/c	C 1 2/c 1
Hall group	-C 2yc	-C 2yc
Moiety formula	2(C57.33 H38 Co2 N1.50 O10), 1.333(C H3) [+ solvent]	C116 H80 Co4 N3 O20
Sum formula	C116 H80 Co4 N3 O20 [+ solvent]	C116 H80 Co4 N3 O20
Mr	2071.54	2071.55
Dx,g cm-3	0.839	0.839
Z	4	4
Mu (mm-1)	0.442	0.442
F000	4260.0	4260.0
F000'	4267.38	
h,k,lmax	39,33,24	39,33,24
Nref	13975	13955
Tmin,Tmax	0.876,0.891	0.637,0.745
Tmin'	0.876	

Correction method= # Reported T Limits: Tmin=0.637 Tmax=0.745
AbsCorr = MULTI-SCAN

Data completeness= 0.999 Theta(max)= 24.713

```
R(reflections)= 0.1022( 7118)                          wR2(reflections)=
                                                       0.3400( 13955)
S = 1.064                      Npar= 693
```

The following ALERTS were generated. Each ALERT has the format
test-name_ALERT_alert-type_alert-level.
Click on the hyperlinks for more details of the test.

Alert level B

```
PLAT934_ALERT_3_B Number of (Iobs-Icalc)/Sigma(W) > 10 Outliers ..          2 Check
```

Alert level C

```
THETM01_ALERT_3_C  The value of sine(theta_max)/wavelength is less than 0.590
            Calculated sin(theta_max)/wavelength =    0.5882
PLAT084_ALERT_3_C High wR2 Value (i.e. > 0.25) ...................       0.34 Report
PLAT094_ALERT_2_C Ratio of Maximum / Minimum Residual Density ....       2.02 Report
PLAT213_ALERT_2_C Atom N1S             has ADP max/min Ratio .....        3.6 prolat
PLAT213_ALERT_2_C Atom C1SB            has ADP max/min Ratio .....        3.2 prolat
PLAT241_ALERT_2_C High   'MainMol' Ueq as Compared to Neighbors of         O1 Check
PLAT241_ALERT_2_C High   'MainMol' Ueq as Compared to Neighbors of        O1S Check
PLAT241_ALERT_2_C High   'MainMol' Ueq as Compared to Neighbors of         O2 Check
PLAT241_ALERT_2_C High   'MainMol' Ueq as Compared to Neighbors of        O2S Check
PLAT241_ALERT_2_C High   'MainMol' Ueq as Compared to Neighbors of        C30 Check
PLAT242_ALERT_2_C Low    'MainMol' Ueq as Compared to Neighbors of        Co1 Check
PLAT242_ALERT_2_C Low    'MainMol' Ueq as Compared to Neighbors of        Co2 Check
PLAT334_ALERT_2_C Small <C-C> Benzene Dist.   C15      -C20      .       1.37 Ang.
PLAT341_ALERT_3_C Low Bond Precision on  C-C Bonds ...............    0.01154 Ang.
PLAT369_ALERT_2_C Long   C(sp2)-C(sp2) Bond  C37       - C41      .       1.53 Ang.
PLAT905_ALERT_3_C Negative K value in the Analysis of Variance ...    -12.852 Report
PLAT910_ALERT_3_C Missing # of FCF Reflection(s) Below Theta(Min).         10 Note
PLAT911_ALERT_3_C Missing FCF Refl Between Thmin & STh/L=    0.588         11 Report
PLAT975_ALERT_2_C Check Calcd Resid. Dens.  1.03Ang From C3S      .       0.51 eA-3
PLAT975_ALERT_2_C Check Calcd Resid. Dens.  0.98Ang From O2       .       0.40 eA-3
```

Alert level G

```
PLAT003_ALERT_2_G Number of Uiso or Uij Restrained non-H Atoms ...         14 Report
PLAT004_ALERT_5_G Polymeric Structure Found with Maximum Dimension          3 Info
PLAT042_ALERT_1_G Calc. and Reported MoietyFormula Strings  Differ     Please Check
PLAT072_ALERT_2_G SHELXL First  Parameter in WGHT  Unusually Large       0.19 Report
PLAT128_ALERT_4_G Alternate Setting for Input Space Group     C2/c       I2/a Note
PLAT171_ALERT_4_G The CIF-Embedded .res File Contains EADP Records          3 Report
PLAT177_ALERT_4_G The CIF-Embedded .res File Contains DELU Records          2 Report
PLAT178_ALERT_4_G The CIF-Embedded .res File Contains SIMU Records          2 Report
PLAT186_ALERT_4_G The CIF-Embedded .res File Contains ISOR Records          2 Report
PLAT199_ALERT_1_G Reported _cell_measurement_temperature ..... (K)        273 Check
PLAT200_ALERT_1_G Reported   _diffrn_ambient_temperature ..... (K)        273 Check
PLAT300_ALERT_4_G Atom Site Occupancy of N1S        Constrained at       0.75 Check
PLAT300_ALERT_4_G Atom Site Occupancy of N2         Constrained at       0.75 Check
PLAT300_ALERT_4_G Atom Site Occupancy of C1SA       Constrained at     0.3333 Check
PLAT300_ALERT_4_G Atom Site Occupancy of C1SB       Constrained at     0.3333 Check
PLAT300_ALERT_4_G Atom Site Occupancy of C2SA       Constrained at     0.3333 Check
PLAT300_ALERT_4_G Atom Site Occupancy of C3S        Constrained at     0.3333 Check
```

```
PLAT300_ALERT_4_G Atom Site Occupancy of C2SB       Constrained at     0.3333 Check
PLAT300_ALERT_4_G Atom Site Occupancy of C5SA       Constrained at     0.3333 Check
PLAT300_ALERT_4_G Atom Site Occupancy of C5SB       Constrained at     0.3333 Check
PLAT300_ALERT_4_G Atom Site Occupancy of C6SA       Constrained at     0.3333 Check
PLAT300_ALERT_4_G Atom Site Occupancy of C6SB       Constrained at     0.3333 Check
PLAT300_ALERT_4_G Atom Site Occupancy of C8S        Constrained at     0.3333 Check
PLAT300_ALERT_4_G Atom Site Occupancy of H2SA       Constrained at     0.3333 Check
PLAT300_ALERT_4_G Atom Site Occupancy of H2SB       Constrained at     0.3333 Check
PLAT300_ALERT_4_G Atom Site Occupancy of H2SC       Constrained at     0.3333 Check
PLAT300_ALERT_4_G Atom Site Occupancy of H2SD       Constrained at     0.3333 Check
PLAT300_ALERT_4_G Atom Site Occupancy of H2SE       Constrained at     0.3333 Check
PLAT300_ALERT_4_G Atom Site Occupancy of H2SF       Constrained at     0.3333 Check
PLAT300_ALERT_4_G Atom Site Occupancy of H3SA       Constrained at     0.3333 Check
PLAT300_ALERT_4_G Atom Site Occupancy of H3SB       Constrained at     0.3333 Check
PLAT300_ALERT_4_G Atom Site Occupancy of H3SC       Constrained at     0.3333 Check
PLAT300_ALERT_4_G Atom Site Occupancy of H6SA       Constrained at     0.3333 Check
PLAT300_ALERT_4_G Atom Site Occupancy of H6SB       Constrained at     0.3333 Check
PLAT300_ALERT_4_G Atom Site Occupancy of H6SC       Constrained at     0.3333 Check
PLAT300_ALERT_4_G Atom Site Occupancy of H6SD       Constrained at     0.3333 Check
PLAT300_ALERT_4_G Atom Site Occupancy of H6SE       Constrained at     0.3333 Check
PLAT300_ALERT_4_G Atom Site Occupancy of H6SF       Constrained at     0.3333 Check
PLAT300_ALERT_4_G Atom Site Occupancy of H8SA       Constrained at     0.3333 Check
PLAT300_ALERT_4_G Atom Site Occupancy of H8SB       Constrained at     0.3333 Check
PLAT300_ALERT_4_G Atom Site Occupancy of H8SC       Constrained at     0.3333 Check
PLAT300_ALERT_4_G Atom Site Occupancy of C4S        Constrained at     0.3333 Check
PLAT300_ALERT_4_G Atom Site Occupancy of H4SA       Constrained at     0.3333 Check
PLAT300_ALERT_4_G Atom Site Occupancy of H4SB       Constrained at     0.3333 Check
PLAT300_ALERT_4_G Atom Site Occupancy of H4SC       Constrained at     0.3333 Check
PLAT300_ALERT_4_G Atom Site Occupancy of C7S        Constrained at     0.3333 Check
PLAT300_ALERT_4_G Atom Site Occupancy of H7SA       Constrained at     0.3333 Check
PLAT300_ALERT_4_G Atom Site Occupancy of H7SB       Constrained at     0.3333 Check
PLAT300_ALERT_4_G Atom Site Occupancy of H7SC       Constrained at     0.3333 Check
PLAT301_ALERT_3_G Main Residue  Disorder .............(Resd  1  )         7% Note
PLAT302_ALERT_4_G Anion/Solvent/Minor-Residue Disorder (Resd  2  )       100% Note
PLAT302_ALERT_4_G Anion/Solvent/Minor-Residue Disorder (Resd  3  )       100% Note
PLAT432_ALERT_2_G Short Inter X...Y Contact  C1SA     ..C4S      .       2.96 Ang.
                                                    x,y,z  =     1_555 Check
PLAT432_ALERT_2_G Short Inter X...Y Contact  C1SB     ..C4S      .       2.36 Ang.
                                                    x,y,z  =     1_555 Check
PLAT432_ALERT_2_G Short Inter X...Y Contact  C3S      ..C4S      .       2.42 Ang.
                                                    x,y,z  =     1_555 Check
PLAT432_ALERT_2_G Short Inter X...Y Contact  C2SB     ..C4S      .       2.80 Ang.
                                                    x,y,z  =     1_555 Check
PLAT432_ALERT_2_G Short Inter X...Y Contact  C5SA     ..C7S      .       2.30 Ang.
                                                    x,y,z  =     1_555 Check
PLAT432_ALERT_2_G Short Inter X...Y Contact  C5SB     ..C7S      .       2.97 Ang.
                                                    x,y,z  =     1_555 Check
PLAT432_ALERT_2_G Short Inter X...Y Contact  C6SA     ..C7S      .       2.73 Ang.
                                                    x,y,z  =     1_555 Check
PLAT432_ALERT_2_G Short Inter X...Y Contact  C7S      ..C8S      .       2.37 Ang.
                                                    x,y,z  =     1_555 Check
PLAT606_ALERT_4_G Solvent Accessible VOID(S) in Structure ........          ! Info
PLAT720_ALERT_4_G Number of Unusual/Non-Standard Labels ..........         32 Note
PLAT764_ALERT_4_G Overcomplete CIF Bond List Detected (Rep/Expd) .       1.16 Ratio
PLAT860_ALERT_3_G Number of Least-Squares Restraints .............        206 Note
PLAT868_ALERT_4_G ALERTS Due to the Use of _smtbx_masks Suppressed          ! Info
PLAT913_ALERT_3_G Missing # of Very Strong Reflections in FCF ....          1 Note
```

```
PLAT930_ALERT_2_G FCF-based   Twin Law ( 1 0 0)         Est.d BASF       0.25 Check
PLAT933_ALERT_2_G Number of HKL-OMIT Records in Embedded .res File          1 Note
PLAT978_ALERT_2_G Number C-C Bonds with Positive Residual Density.          0 Info
```

```
   0 ALERT level A = Most likely a serious problem - resolve or explain
   1 ALERT level B = A potentially serious problem, consider carefully
  20 ALERT level C = Check. Ensure it is not caused by an omission or oversight
  69 ALERT level G = General information/check it is not something unexpected

   3 ALERT type 1 CIF construction/syntax error, inconsistent or missing data
  27 ALERT type 2 Indicator that the structure model may be wrong or deficient
  10 ALERT type 3 Indicator that the structure quality may be low
  49 ALERT type 4 Improvement, methodology, query or suggestion
   1 ALERT type 5 Informative message, check
```

It is advisable to attempt to resolve as many as possible of the alerts in all categories. Often the minor alerts point to easily fixed oversights, errors and omissions in your CIF or refinement strategy, so attention to these fine details can be worthwhile. In order to resolve some of the more serious problems it may be necessary to carry out additional measurements or structure refinements. However, the purpose of your study may justify the reported deviations and the more serious of these should normally be commented upon in the discussion or experimental section of a paper or in the "special_details" fields of the CIF. checkCIF was carefully designed to identify outliers and unusual parameters, but every test has its limitations and alerts that are not important in a particular case may appear. Conversely, the absence of alerts does not guarantee there are no aspects of the results needing attention. It is up to the individual to critically assess their own results and, if necessary, seek expert advice.

Publication of your CIF in IUCr journals

A basic structural check has been run on your CIF. These basic checks will be run on all CIFs submitted for publication in IUCr journals (*Acta Crystallographica*, *Journal of Applied Crystallography*, *Journal of Synchrotron Radiation*); however, if you intend to submit to *Acta Crystallographica Section C* or *E* or *IUCrData*, you should make sure that full publication checks are run on the final version of your CIF prior to submission.

Publication of your CIF in other journals

Please refer to the *Notes for Authors* of the relevant journal for any special instructions relating to CIF submission.

PLATON version of 06/07/2023; check.def file version of 30/06/2023

Datablock mjh_co_mettc_0m - ellipsoid plot

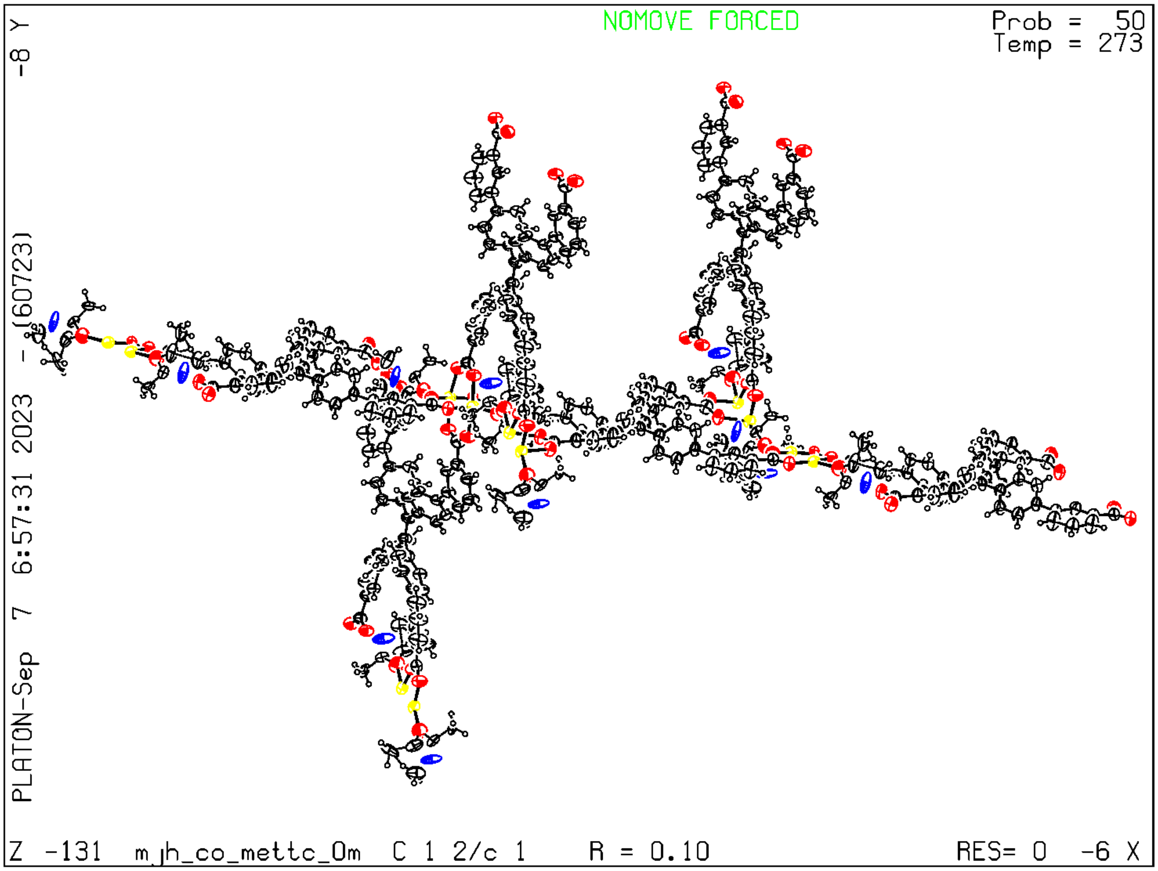

Supplement: Supplementary file 4 [file DataSheet6.PDF]
