# Supplementary material for: Probing ligand conformation and net dimensionality in a series of tetraphenylethene-based metal–organic frameworks
Source: Front Chem. 2024 Apr 25;12:1396123. doi: 10.3389/fchem.2024.1396123 (PMC11079141; doi:10.3389/fchem.2024.1396123)

checkCIF/PLATON report

Structure factors have been supplied for datablock(s) wsu11-zn

THIS REPORT IS FOR GUIDANCE ONLY. IF USED AS PART OF A REVIEW PROCEDURE FOR PUBLICATION, IT SHOULD NOT REPLACE THE EXPERTISE OF AN EXPERIENCED CRYSTALLOGRAPHIC REFEREE.

No syntax errors found. CIF dictionary Interpreting this report

Datablock: wsu11-zn

Bond precision:	C-C = 0.0050 A		Wavelength=0.71073
Cell:	a=19.5604(11)	b=19.5604(11)	c=21.8824(14)
	alpha=90	beta=90	gamma=90
Temperature:	273 K		

	Calculated	Reported
Volume	8372.4(11)	8372.4(11)
Space group	P 4/n n c	P 4/n n c
Hall group	-P 4a 2bc	-P 4a 2bc
Moiety formula	C56 H34 N O9 Zn2 [+ solvent]	C56 H34 N O9 Zn2
Sum formula	C56 H34 N O9 Zn2 [+ solvent]	C56 H34 N O9 Zn2
Mr	995.62	995.58
Dx,g cm-3	0.790	0.790
Z	4	4
Mu (mm-1)	0.607	0.607
F000	2036.0	2036.0
F000'	2039.11	
h,k,lmax	23,23,25	22,23,25
Nref	3597	3593
Tmin,Tmax	0.804,0.988	0.804,0.988
Tmin'	0.804	

Correction method= # Reported T Limits: Tmin=0.804 Tmax=0.988
AbsCorr = MULTI-SCAN

Data completeness= 0.999 Theta(max)= 24.733

R(reflections)= 0.0482(2300)   wR2(reflections)= 0.1807(3593)

S = 1.090   Npar= 160

The following ALERTS were generated. Each ALERT has the format
test-name_ALERT_alert-type_alert-level.
Click on the hyperlinks for more details of the test.

Alert level B

```
PLAT242_ALERT_2_B Low    'MainMol' Ueq as Compared to Neighbors of        Zn2 Check
```

Alert level C

```
CRYSC01_ALERT_1_C The word below has not been recognised as a standard
            identifier.
            yellowish
THETM01_ALERT_3_C  The value of sine(theta_max)/wavelength is less than 0.590
            Calculated sin(theta_max)/wavelength =    0.5887
PLAT218_ALERT_3_C Constrained U(ij) Components(s) for C15        .          3 Check
PLAT218_ALERT_3_C Constrained U(ij) Components(s) for C15A       .          3 Check
PLAT241_ALERT_2_C High   'MainMol' Ueq as Compared to Neighbors of         O1 Check
PLAT241_ALERT_2_C High   'MainMol' Ueq as Compared to Neighbors of         O2 Check
PLAT242_ALERT_2_C Low    'MainMol' Ueq as Compared to Neighbors of        Zn1 Check
PLAT767_ALERT_4_C INS Embedded LIST 6 Instruction Should be LIST 4     Please Check
PLAT905_ALERT_3_C Negative K value in the Analysis of Variance ...    -24.704 Report
PLAT905_ALERT_3_C Negative K value in the Analysis of Variance ...     -1.253 Report
PLAT918_ALERT_3_C Reflection(s) with I(obs) much Smaller I(calc) .          1 Check
PLAT934_ALERT_3_C Number of (Iobs-Icalc)/Sigma(W) > 10 Outliers ..          1 Check
```

Alert level G

```
PLAT002_ALERT_2_G Number of Distance or Angle Restraints on AtSite          3 Note
PLAT004_ALERT_5_G Polymeric Structure Found with Maximum Dimension          3 Info
PLAT066_ALERT_1_G Predicted and Reported Tmin&Tmax Range Identical          ? Check
PLAT072_ALERT_2_G SHELXL First  Parameter in WGHT  Unusually Large       0.11 Report
PLAT171_ALERT_4_G The CIF-Embedded .res File Contains EADP Records          2 Report
PLAT176_ALERT_4_G The CIF-Embedded .res File Contains SADI Records          1 Report
PLAT199_ALERT_1_G Reported _cell_measurement_temperature ..... (K)        273 Check
PLAT200_ALERT_1_G Reported   _diffrn_ambient_temperature ..... (K)        273 Check
PLAT301_ALERT_3_G Main Residue  Disorder .............(Resd  1  )          3% Note
PLAT432_ALERT_2_G Short Inter X...Y Contact  N1       ..C15      .       2.36 Ang.
                                   3/2-y,3/2-x,1/2-z  =      8_665 Check
PLAT432_ALERT_2_G Short Inter X...Y Contact  N1       ..C15      .       2.36 Ang.
                                       3/2-x,y,1/2-z  =      5_655 Check
PLAT432_ALERT_2_G Short Inter X...Y Contact  N1       ..C15A     .       2.41 Ang.
                                   3/2-y,3/2-x,1/2-z  =      8_665 Check
PLAT432_ALERT_2_G Short Inter X...Y Contact  N1       ..C15A     .       2.41 Ang.
                                       3/2-x,y,1/2-z  =      5_655 Check
PLAT606_ALERT_4_G Solvent Accessible VOID(S) in Structure ........          ! Info
PLAT794_ALERT_5_G Tentative Bond Valency for Zn2        (II)     .       2.12 Info
PLAT860_ALERT_3_G Number of Least-Squares Restraints ............          1 Note
PLAT868_ALERT_4_G ALERTS Due to the Use of _smtbx_masks Suppressed          ! Info
PLAT909_ALERT_3_G Percentage of I>2sig(I) Data at Theta(Max) Still        30% Note
PLAT910_ALERT_3_G Missing # of FCF Reflection(s) Below Theta(Min).          4 Note
```

```
PLAT913_ALERT_3_G Missing # of Very Strong Reflections in FCF ....          2 Note
PLAT933_ALERT_2_G Number of HKL-OMIT Records in Embedded .res File          1 Note
PLAT978_ALERT_2_G Number C-C Bonds with Positive Residual Density.          1 Info
```

```
   0 ALERT level A = Most likely a serious problem - resolve or explain
   1 ALERT level B = A potentially serious problem, consider carefully
  12 ALERT level C = Check. Ensure it is not caused by an omission or oversight
  22 ALERT level G = General information/check it is not something unexpected

   4 ALERT type 1 CIF construction/syntax error, inconsistent or missing data
  12 ALERT type 2 Indicator that the structure model may be wrong or deficient
  12 ALERT type 3 Indicator that the structure quality may be low
   5 ALERT type 4 Improvement, methodology, query or suggestion
   2 ALERT type 5 Informative message, check
```

It is advisable to attempt to resolve as many as possible of the alerts in all categories. Often the minor alerts point to easily fixed oversights, errors and omissions in your CIF or refinement strategy, so attention to these fine details can be worthwhile. In order to resolve some of the more serious problems it may be necessary to carry out additional measurements or structure refinements. However, the purpose of your study may justify the reported deviations and the more serious of these should normally be commented upon in the discussion or experimental section of a paper or in the "special_details" fields of the CIF. checkCIF was carefully designed to identify outliers and unusual parameters, but every test has its limitations and alerts that are not important in a particular case may appear. Conversely, the absence of alerts does not guarantee there are no aspects of the results needing attention. It is up to the individual to critically assess their own results and, if necessary, seek expert advice.

Publication of your CIF in IUCr journals

A basic structural check has been run on your CIF. These basic checks will be run on all CIFs submitted for publication in IUCr journals (*Acta Crystallographica*, *Journal of Applied Crystallography*, *Journal of Synchrotron Radiation*); however, if you intend to submit to *Acta Crystallographica Section C* or *E* or *IUCrData*, you should make sure that full publication checks are run on the final version of your CIF prior to submission.

Publication of your CIF in other journals

Please refer to the *Notes for Authors* of the relevant journal for any special instructions relating to CIF submission.

PLATON version of 06/07/2023; check.def file version of 30/06/2023

Datablock wsu11-zn - ellipsoid plot

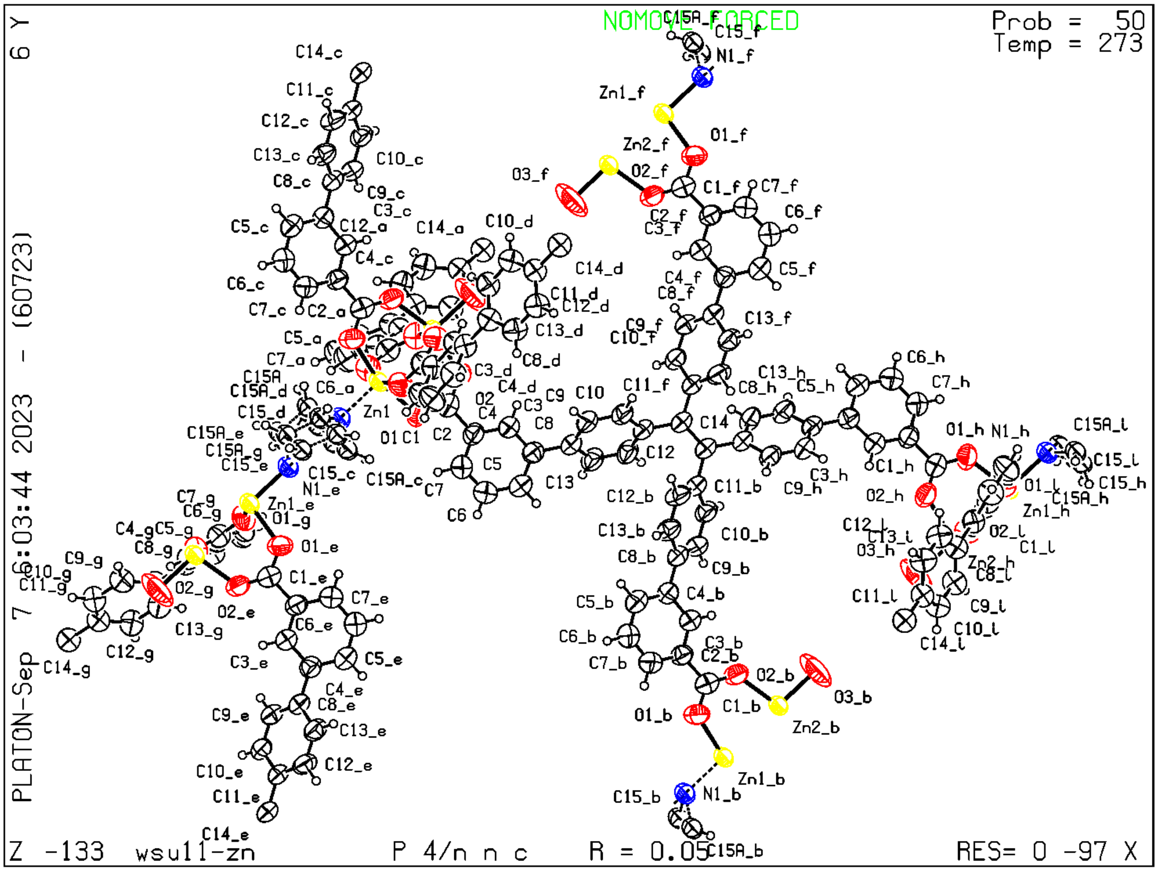

Supplement: Supplementary file 5 [file DataSheet3.PDF]
