# Supplementary material for: Probing ligand conformation and net dimensionality in a series of tetraphenylethene-based metal–organic frameworks
Source: Front Chem. 2024 Apr 25;12:1396123. doi: 10.3389/fchem.2024.1396123 (PMC11079141; doi:10.3389/fchem.2024.1396123)

checkCIF/PLATON report

Structure factors have been supplied for datablock(s) wsu10-cu

THIS REPORT IS FOR GUIDANCE ONLY. IF USED AS PART OF A REVIEW PROCEDURE FOR PUBLICATION, IT SHOULD NOT REPLACE THE EXPERTISE OF AN EXPERIENCED CRYSTALLOGRAPHIC REFEREE.

No syntax errors found. CIF dictionary Interpreting this report

Datablock: wsu10-cu

Bond precision:	C-C = 0.0219 A		Wavelength=0.71073
Cell:	a=19.3354(19)	b=19.3354(19)	c=20.825(2)
	alpha=90	beta=90	gamma=90
Temperature:	273 K		

	Calculated	Reported
Volume	7785.6(17)	7785.5(17)
Space group	P 4/n n c	P 4/n n c
Hall group	-P 4a 2bc	-P 4a 2bc
Moiety formula	C54 H32 Cu2 O9 [+ solvent]	C54 H32 Cu2 O9
Sum formula	C54 H32 Cu2 O9 [+ solvent]	C54 H32 Cu2 O9
Mr	951.90	951.87
Dx,g cm-3	0.812	0.812
Z	4	4
Mu (mm-1)	0.580	0.580
F000	1944.0	1944.0
F000'	1947.37	
h,k,lmax	15,15,16	15,15,16
Nref	1066	1060
Tmin,Tmax	0.944,0.988	0.944,0.988
Tmin'	0.944	

Correction method= # Reported T Limits: Tmin=0.944 Tmax=0.988
AbsCorr = MULTI-SCAN

Data completeness= 0.994 Theta(max)= 16.553

R(reflections)= 0.0810(937) wR2(reflections)= 0.2463(1060)

S = 1.115 Npar= 150

The following ALERTS were generated. Each ALERT has the format
test-name_ALERT_alert-type_alert-level.
Click on the hyperlinks for more details of the test.

Alert level A

THETM01_ALERT_3_A The value of sine(theta_max)/wavelength is less than 0.550
Calculated sin(theta_max)/wavelength = 0.4009
MOF materials are usually diffract weakly at higher angles.

Alert level B

PLAT088_ALERT_3_B Poor Data / Parameter Ratio .... 7.07 Note
PLAT094_ALERT_2_B Ratio of Maximum / Minimum Residual Density .... 4.14 Report
PLAT242_ALERT_2_B Low 'MainMol' Ueq as Compared to Neighbors of Cu1 Check
PLAT341_ALERT_3_B Low Bond Precision on C-C Bonds .... 0.02187 Ang.

Alert level C

PLAT234_ALERT_4_C Large Hirshfeld Difference Cu1 --O1 . 0.21 Ang.
PLAT234_ALERT_4_C Large Hirshfeld Difference O2 --C1 . 0.24 Ang.
PLAT234_ALERT_4_C Large Hirshfeld Difference C6 --C8 . 0.18 Ang.
PLAT241_ALERT_2_C High 'MainMol' Ueq as Compared to Neighbors of O2 Check
PLAT241_ALERT_2_C High 'MainMol' Ueq as Compared to Neighbors of O3 Check
PLAT250_ALERT_2_C Large U3/U1 Ratio for Average U(i,j) Tensor .... 2.5 Note
PLAT260_ALERT_2_C Large Average Ueq of Residue Including Cu1 0.122 Check
PLAT334_ALERT_2_C Small <C-C> Benzene Dist. C2 -C7 . 1.37 Ang.
PLAT767_ALERT_4_C INS Embedded LIST 6 Instruction Should be LIST 4 Please Check
PLAT906_ALERT_3_C Large K Value in the Analysis of Variance ...... 2.127 Check
PLAT910_ALERT_3_C Missing # of FCF Reflection(s) Below Theta(Min). 6 Note
PLAT918_ALERT_3_C Reflection(s) with I(obs) much Smaller I(calc) . 4 Check
PLAT922_ALERT_1_C wR2 in the CIF and FCF Differ by .... 0.0025 Check
PLAT923_ALERT_1_C S Values in the CIF and FCF Differ by .... 0.013 Check

Alert level G

PLAT003_ALERT_2_G Number of Uiso or Uij Restrained non-H Atoms ... 13 Report
PLAT004_ALERT_5_G Polymeric Structure Found with Maximum Dimension 2 Info
PLAT066_ALERT_1_G Predicted and Reported Tmin&Tmax Range Identical ? Check
PLAT072_ALERT_2_G SHELXL First Parameter in WGHT Unusually Large 0.14 Report
PLAT083_ALERT_2_G SHELXL Second Parameter in WGHT Unusually Large 32.27 Why ?
PLAT187_ALERT_4_G The CIF-Embedded .res File Contains RIGU Records 2 Report
PLAT199_ALERT_1_G Reported _cell_measurement_temperature ..... (K) 273 Check
PLAT200_ALERT_1_G Reported _diffrn_ambient_temperature ..... (K) 273 Check
PLAT606_ALERT_4_G Solvent Accessible VOID(S) in Structure .... ! Info
PLAT794_ALERT_5_G Tentative Bond Valency for Cu1 (II) . 2.29 Info
PLAT860_ALERT_3_G Number of Least-Squares Restraints .... 81 Note
PLAT868_ALERT_4_G ALERTS Due to the Use of _smtbx_masks Suppressed ! Info
PLAT909_ALERT_3_G Percentage of I>2sig(I) Data at Theta(Max) Still 78% Note
PLAT913_ALERT_3_G Missing # of Very Strong Reflections in FCF .... 3 Note
PLAT933_ALERT_2_G Number of HKL-OMIT Records in Embedded .res File 1 Note
PLAT978_ALERT_2_G Number C-C Bonds with Positive Residual Density. 0 Info

1 **ALERT level A** = Most likely a serious problem - resolve or explain
4 **ALERT level B** = A potentially serious problem, consider carefully

```
14 ALERT level C = Check. Ensure it is not caused by an omission or oversight
16 ALERT level G = General information/check it is not something unexpected

 5 ALERT type 1 CIF construction/syntax error, inconsistent or missing data
12 ALERT type 2 Indicator that the structure model may be wrong or deficient
 9 ALERT type 3 Indicator that the structure quality may be low
 7 ALERT type 4 Improvement, methodology, query or suggestion
 2 ALERT type 5 Informative message, check
```

It is advisable to attempt to resolve as many as possible of the alerts in all categories. Often the minor alerts point to easily fixed oversights, errors and omissions in your CIF or refinement strategy, so attention to these fine details can be worthwhile. In order to resolve some of the more serious problems it may be necessary to carry out additional measurements or structure refinements. However, the purpose of your study may justify the reported deviations and the more serious of these should normally be commented upon in the discussion or experimental section of a paper or in the "special_details" fields of the CIF. checkCIF was carefully designed to identify outliers and unusual parameters, but every test has its limitations and alerts that are not important in a particular case may appear. Conversely, the absence of alerts does not guarantee there are no aspects of the results needing attention. It is up to the individual to critically assess their own results and, if necessary, seek expert advice.

Publication of your CIF in IUCr journals

A basic structural check has been run on your CIF. These basic checks will be run on all CIFs submitted for publication in IUCr journals (*Acta Crystallographica*, *Journal of Applied Crystallography*, *Journal of Synchrotron Radiation*); however, if you intend to submit to *Acta Crystallographica Section C* or *E* or *IUCrData*, you should make sure that full publication checks are run on the final version of your CIF prior to submission.

Publication of your CIF in other journals

Please refer to the *Notes for Authors* of the relevant journal for any special instructions relating to CIF submission.

PLATON version of 06/07/2023; check.def file version of 30/06/2023

Datablock wsu10-cu - ellipsoid plot

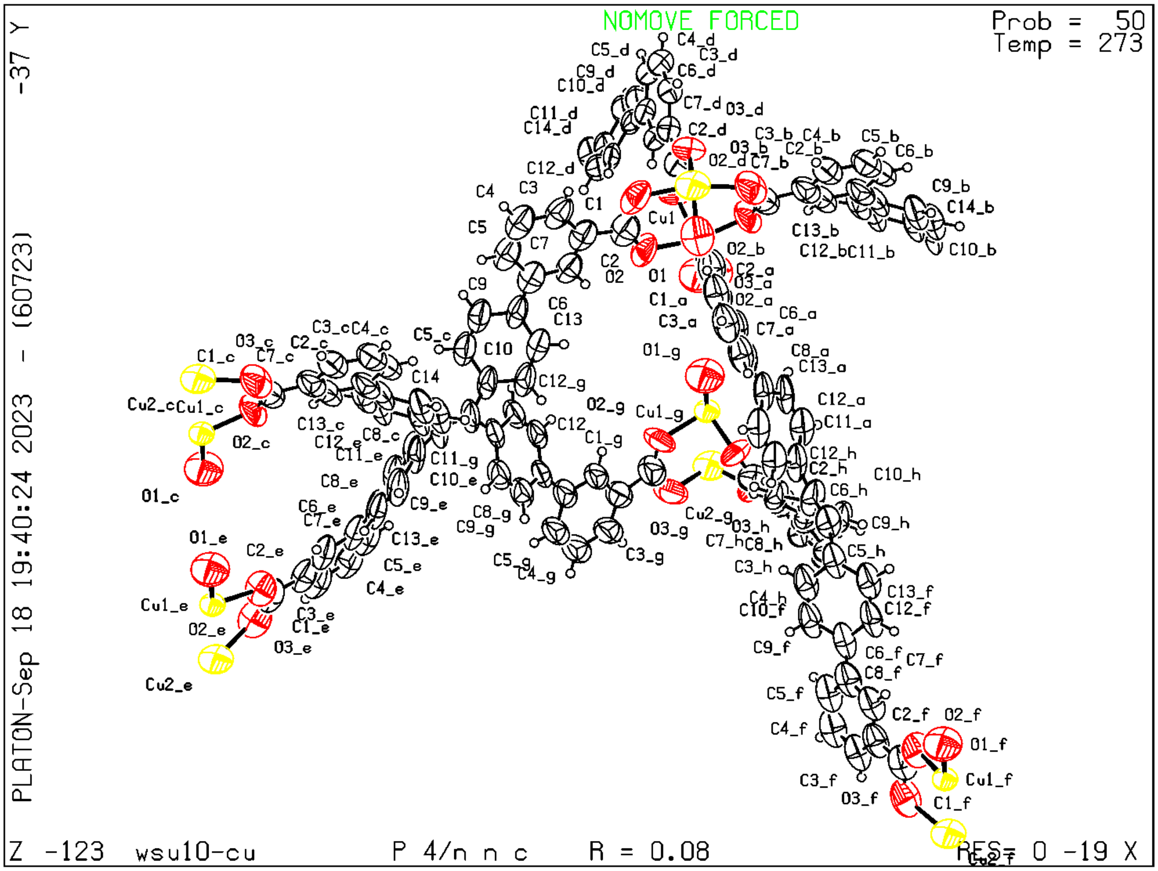

Supplement: Supplementary file 6 [file DataSheet1.PDF]
