# Supplementary material for: Probing ligand conformation and net dimensionality in a series of tetraphenylethene-based metal–organic frameworks
Source: Front Chem. 2024 Apr 25;12:1396123. doi: 10.3389/fchem.2024.1396123 (PMC11079141; doi:10.3389/fchem.2024.1396123)

checkCIF/PLATON report

Structure factors have been supplied for datablock(s) wsu20-zn

THIS REPORT IS FOR GUIDANCE ONLY. IF USED AS PART OF A REVIEW PROCEDURE FOR PUBLICATION, IT SHOULD NOT REPLACE THE EXPERTISE OF AN EXPERIENCED CRYSTALLOGRAPHIC REFEREE.

No syntax errors found.   CIF dictionary   Interpreting this report

Datablock: wsu20-zn

Bond precision:	C-C = 0.0082 A		Wavelength=0.71073
Cell:	a=33.730(3)	b=28.276(3)	c=19.9896(18)
	alpha=90	beta=122.708(3)	gamma=90
Temperature:	273 K		

	Calculated	Reported
Volume	16042(3)	16042(3)
Space group	C 2/c	C 1 2/c 1
Hall group	-C 2yc	-C 2yc
Moiety formula	C62 H50 N2 O10 Zn2 [+ solvent]	C62 H50 N2 O10 Zn2
Sum formula	C62 H50 N2 O10 Zn2 [+ solvent]	C62 H50 N2 O10 Zn2
Mr	1113.82	1113.78
Dx,g cm-3	0.922	0.922
Z	8	8
Mu (mm-1)	0.640	0.640
F000	4608.0	4608.0
F000’	4614.40	
h,k,lmax	39,33,23	39,33,23
Nref	13748	13655
Tmin,Tmax	0.891,0.908	0.891,0.908
Tmin’	0.852	

Correction method= # Reported T Limits: Tmin=0.891 Tmax=0.908
AbsCorr = MULTI-SCAN

Data completeness= 0.993   Theta(max)= 24.750

R(reflections)= 0.0727(9611) wR2(reflections)= 0.2439(13655)

S = 1.066 Npar= 597

The following ALERTS were generated. Each ALERT has the format
test-name_ALERT_alert-type_alert-level.
Click on the hyperlinks for more details of the test.

Alert level B

```
PLAT910_ALERT_3_B Missing # of FCF Reflection(s) Below Theta(Min).         11 Note
```

Alert level C

```
THETM01_ALERT_3_C  The value of sine(theta_max)/wavelength is less than 0.590
            Calculated sin(theta_max)/wavelength =    0.5891
PLAT218_ALERT_3_C Constrained U(ij) Components(s) for N1A        .          6 Check
PLAT218_ALERT_3_C Constrained U(ij) Components(s) for N1S        .          6 Check
PLAT218_ALERT_3_C Constrained U(ij) Components(s) for N2S        .          6 Check
PLAT218_ALERT_3_C Constrained U(ij) Components(s) for N5         .          6 Check
PLAT218_ALERT_3_C Constrained U(ij) Components(s) for C1SA       .          6 Check
PLAT218_ALERT_3_C Constrained U(ij) Components(s) for C1SB       .          6 Check
PLAT218_ALERT_3_C Constrained U(ij) Components(s) for C2SA       .          6 Check
PLAT218_ALERT_3_C Constrained U(ij) Components(s) for C2SB       .          6 Check
PLAT218_ALERT_3_C Constrained U(ij) Components(s) for C3SA       .          6 Check
PLAT218_ALERT_3_C Constrained U(ij) Components(s) for C3SB       .          6 Check
PLAT218_ALERT_3_C Constrained U(ij) Components(s) for C4SA       .          6 Check
PLAT218_ALERT_3_C Constrained U(ij) Components(s) for C4SB       .          6 Check
PLAT218_ALERT_3_C Constrained U(ij) Components(s) for C5SA       .          6 Check
PLAT218_ALERT_3_C Constrained U(ij) Components(s) for C5SB       .          6 Check
PLAT218_ALERT_3_C Constrained U(ij) Components(s) for C6SA       .          6 Check
PLAT218_ALERT_3_C Constrained U(ij) Components(s) for C6SB       .          6 Check
PLAT218_ALERT_3_C Constrained U(ij) Components(s) for C7SA       .          6 Check
PLAT218_ALERT_3_C Constrained U(ij) Components(s) for C7SB       .          6 Check
PLAT218_ALERT_3_C Constrained U(ij) Components(s) for C8SA       .          6 Check
PLAT218_ALERT_3_C Constrained U(ij) Components(s) for C8SB       .          6 Check
PLAT241_ALERT_2_C High   'MainMol' Ueq as Compared to Neighbors of         O6 Check
PLAT241_ALERT_2_C High   'MainMol' Ueq as Compared to Neighbors of         C4 Check
PLAT242_ALERT_2_C Low    'MainMol' Ueq as Compared to Neighbors of        Zn1 Check
PLAT242_ALERT_2_C Low    'MainMol' Ueq as Compared to Neighbors of        Zn2 Check
PLAT341_ALERT_3_C Low Bond Precision on  C-C Bonds ..............    0.00816 Ang.
PLAT767_ALERT_4_C INS Embedded LIST 6 Instruction Should be LIST 4     Please Check
PLAT905_ALERT_3_C Negative K value in the Analysis of Variance ...     -2.729 Report
PLAT911_ALERT_3_C Missing FCF Refl Between Thmin & STh/L=    0.589         83 Report
PLAT918_ALERT_3_C Reflection(s) with I(obs) much Smaller I(calc) .          1 Check
PLAT934_ALERT_3_C Number of (Iobs-Icalc)/Sigma(W) > 10 Outliers ..          1 Check
PLAT975_ALERT_2_C Check Calcd Resid. Dens.  1.06Ang From O1S     .       1.32 eA-3
PLAT977_ALERT_2_C Check Negative Difference Density on H7SA      .      -0.39 eA-3
```

Alert level G

```
PLAT002_ALERT_2_G Number of Distance or Angle Restraints on AtSite         22 Note
PLAT004_ALERT_5_G Polymeric Structure Found with Maximum Dimension          3 Info
PLAT066_ALERT_1_G Predicted and Reported Tmin&Tmax Range Identical          ? Check
PLAT072_ALERT_2_G SHELXL First  Parameter in WGHT  Unusually Large       0.15 Report
```

```
PLAT083_ALERT_2_G SHELXL Second Parameter in WGHT  Unusually Large      15.95 Why ?
PLAT128_ALERT_4_G Alternate Setting for Input Space Group     C2/c       I2/a Note
PLAT169_ALERT_4_G The CIF-Embedded .res File Contains AFIX 1 Recds         56 Report
PLAT172_ALERT_4_G The CIF-Embedded .res File Contains DFIX Records         14 Report
PLAT173_ALERT_4_G The CIF-Embedded .res File Contains DANG Records          6 Report
PLAT174_ALERT_4_G The CIF-Embedded .res File Contains FLAT Records          2 Report
PLAT176_ALERT_4_G The CIF-Embedded .res File Contains SADI Records         22 Report
PLAT191_ALERT_3_G A Non-default SADI Restraint Value has been used     0.0400 Report
PLAT191_ALERT_3_G A Non-default SADI Restraint Value has been used     0.0400 Report
PLAT191_ALERT_3_G A Non-default SADI Restraint Value has been used     0.0400 Report
PLAT191_ALERT_3_G A Non-default SADI Restraint Value has been used     0.0400 Report
PLAT191_ALERT_3_G A Non-default SADI Restraint Value has been used     0.0400 Report
PLAT191_ALERT_3_G A Non-default SADI Restraint Value has been used     0.0400 Report
PLAT191_ALERT_3_G A Non-default SADI Restraint Value has been used     0.0400 Report
PLAT191_ALERT_3_G A Non-default SADI Restraint Value has been used     0.0400 Report
PLAT191_ALERT_3_G A Non-default SADI Restraint Value has been used     0.0400 Report
PLAT191_ALERT_3_G A Non-default SADI Restraint Value has been used     0.0400 Report
PLAT191_ALERT_3_G A Non-default SADI Restraint Value has been used     0.0400 Report
PLAT191_ALERT_3_G A Non-default SADI Restraint Value has been used     0.0400 Report
PLAT199_ALERT_1_G Reported _cell_measurement_temperature ..... (K)        273 Check
PLAT200_ALERT_1_G Reported   _diffrn_ambient_temperature ..... (K)        273 Check
PLAT300_ALERT_4_G Atom Site Occupancy of N2S        Constrained at       0.53 Check
PLAT300_ALERT_4_G Atom Site Occupancy of N5         Constrained at     0.5071 Check
PLAT300_ALERT_4_G Atom Site Occupancy of C1SA       Constrained at     0.5071 Check
PLAT300_ALERT_4_G Atom Site Occupancy of C2SA       Constrained at     0.5071 Check
PLAT300_ALERT_4_G Atom Site Occupancy of C3SB       Constrained at     0.5071 Check
PLAT300_ALERT_4_G Atom Site Occupancy of C4SA       Constrained at     0.5071 Check
PLAT300_ALERT_4_G Atom Site Occupancy of C5SB       Constrained at       0.53 Check
PLAT300_ALERT_4_G Atom Site Occupancy of C6SB       Constrained at       0.53 Check
PLAT300_ALERT_4_G Atom Site Occupancy of C7SA       Constrained at       0.53 Check
PLAT300_ALERT_4_G Atom Site Occupancy of C8SA       Constrained at       0.53 Check
PLAT300_ALERT_4_G Atom Site Occupancy of N1A        Constrained at       0.47 Check
PLAT300_ALERT_4_G Atom Site Occupancy of N1S        Constrained at     0.4929 Check
PLAT300_ALERT_4_G Atom Site Occupancy of C1SB       Constrained at     0.4929 Check
PLAT300_ALERT_4_G Atom Site Occupancy of C2SB       Constrained at     0.4929 Check
PLAT300_ALERT_4_G Atom Site Occupancy of C3SA       Constrained at     0.4929 Check
PLAT300_ALERT_4_G Atom Site Occupancy of C4SB       Constrained at     0.4929 Check
PLAT300_ALERT_4_G Atom Site Occupancy of C5SA       Constrained at       0.47 Check
PLAT300_ALERT_4_G Atom Site Occupancy of C6SA       Constrained at       0.47 Check
PLAT300_ALERT_4_G Atom Site Occupancy of C7SB       Constrained at       0.47 Check
PLAT300_ALERT_4_G Atom Site Occupancy of C8SB       Constrained at       0.47 Check
PLAT300_ALERT_4_G Atom Site Occupancy of H2SA       Constrained at     0.5071 Check
PLAT300_ALERT_4_G Atom Site Occupancy of H2SB       Constrained at     0.5071 Check
PLAT300_ALERT_4_G Atom Site Occupancy of H2SC       Constrained at     0.5071 Check
PLAT300_ALERT_4_G Atom Site Occupancy of H3SD       Constrained at     0.5071 Check
PLAT300_ALERT_4_G Atom Site Occupancy of H3SE       Constrained at     0.5071 Check
PLAT300_ALERT_4_G Atom Site Occupancy of H3SF       Constrained at     0.5071 Check
PLAT300_ALERT_4_G Atom Site Occupancy of H4SA       Constrained at     0.5071 Check
PLAT300_ALERT_4_G Atom Site Occupancy of H4SB       Constrained at     0.5071 Check
PLAT300_ALERT_4_G Atom Site Occupancy of H4SC       Constrained at     0.5071 Check
PLAT300_ALERT_4_G Atom Site Occupancy of H6SD       Constrained at       0.53 Check
PLAT300_ALERT_4_G Atom Site Occupancy of H6SE       Constrained at       0.53 Check
PLAT300_ALERT_4_G Atom Site Occupancy of H6SF       Constrained at       0.53 Check
PLAT300_ALERT_4_G Atom Site Occupancy of H7SA       Constrained at       0.53 Check
PLAT300_ALERT_4_G Atom Site Occupancy of H7SB       Constrained at       0.53 Check
PLAT300_ALERT_4_G Atom Site Occupancy of H7SC       Constrained at       0.53 Check
PLAT300_ALERT_4_G Atom Site Occupancy of H8SA       Constrained at       0.53 Check
```

```
PLAT300_ALERT_4_G Atom Site Occupancy of H8SB       Constrained at       0.53 Check
PLAT300_ALERT_4_G Atom Site Occupancy of H8SC       Constrained at       0.53 Check
PLAT300_ALERT_4_G Atom Site Occupancy of H2SD       Constrained at     0.4929 Check
PLAT300_ALERT_4_G Atom Site Occupancy of H2SE       Constrained at     0.4929 Check
PLAT300_ALERT_4_G Atom Site Occupancy of H2SF       Constrained at     0.4929 Check
PLAT300_ALERT_4_G Atom Site Occupancy of H3SA       Constrained at     0.4929 Check
PLAT300_ALERT_4_G Atom Site Occupancy of H3SB       Constrained at     0.4929 Check
PLAT300_ALERT_4_G Atom Site Occupancy of H3SC       Constrained at     0.4929 Check
PLAT300_ALERT_4_G Atom Site Occupancy of H4SD       Constrained at     0.4929 Check
PLAT300_ALERT_4_G Atom Site Occupancy of H4SE       Constrained at     0.4929 Check
PLAT300_ALERT_4_G Atom Site Occupancy of H4SF       Constrained at     0.4929 Check
PLAT300_ALERT_4_G Atom Site Occupancy of H6SA       Constrained at       0.47 Check
PLAT300_ALERT_4_G Atom Site Occupancy of H6SB       Constrained at       0.47 Check
PLAT300_ALERT_4_G Atom Site Occupancy of H6SC       Constrained at       0.47 Check
PLAT300_ALERT_4_G Atom Site Occupancy of H7SD       Constrained at       0.47 Check
PLAT300_ALERT_4_G Atom Site Occupancy of H7SE       Constrained at       0.47 Check
PLAT300_ALERT_4_G Atom Site Occupancy of H7SF       Constrained at       0.47 Check
PLAT300_ALERT_4_G Atom Site Occupancy of H8SD       Constrained at       0.47 Check
PLAT300_ALERT_4_G Atom Site Occupancy of H8SE       Constrained at       0.47 Check
PLAT300_ALERT_4_G Atom Site Occupancy of H8SF       Constrained at       0.47 Check
PLAT301_ALERT_3_G Main Residue  Disorder .............(Resd  1  )         13% Note
PLAT606_ALERT_4_G Solvent Accessible VOID(S) in Structure ........          ! Info
PLAT720_ALERT_4_G Number of Unusual/Non-Standard Labels ..........         52 Note
PLAT789_ALERT_4_G Atoms with Negative _atom_site_disorder_group  #         56 Check
PLAT822_ALERT_4_G CIF-embedded .res Contains Negative PART Numbers         18 Check
PLAT860_ALERT_3_G Number of Least-Squares Restraints ............          52 Note
PLAT868_ALERT_4_G ALERTS Due to the Use of _smtbx_masks Suppressed          ! Info
PLAT909_ALERT_3_G Percentage of I>2sig(I) Data at Theta(Max) Still        41% Note
PLAT913_ALERT_3_G Missing # of Very Strong Reflections in FCF ....          3 Note
PLAT933_ALERT_2_G Number of HKL-OMIT Records in Embedded .res File         12 Note
PLAT978_ALERT_2_G Number C-C Bonds with Positive Residual Density.          0 Info
```

```
   0 ALERT level A = Most likely a serious problem - resolve or explain
   1 ALERT level B = A potentially serious problem, consider carefully
  33 ALERT level C = Check. Ensure it is not caused by an omission or oversight
  92 ALERT level G = General information/check it is not something unexpected

   3 ALERT type 1 CIF construction/syntax error, inconsistent or missing data
  11 ALERT type 2 Indicator that the structure model may be wrong or deficient
  43 ALERT type 3 Indicator that the structure quality may be low
  68 ALERT type 4 Improvement, methodology, query or suggestion
   1 ALERT type 5 Informative message, check
```

It is advisable to attempt to resolve as many as possible of the alerts in all categories. Often the minor alerts point to easily fixed oversights, errors and omissions in your CIF or refinement strategy, so attention to these fine details can be worthwhile. In order to resolve some of the more serious problems it may be necessary to carry out additional measurements or structure refinements. However, the purpose of your study may justify the reported deviations and the more serious of these should normally be commented upon in the discussion or experimental section of a paper or in the "special_details" fields of the CIF. checkCIF was carefully designed to identify outliers and unusual parameters, but every test has its limitations and alerts that are not important in a particular case may appear. Conversely, the absence of alerts does not guarantee there are no aspects of the results needing attention. It is up to the individual to critically assess their own results and, if necessary, seek expert advice.

Publication of your CIF in IUCr journals

A basic structural check has been run on your CIF. These basic checks will be run on all CIFs submitted for publication in IUCr journals (*Acta Crystallographica*, *Journal of Applied Crystallography*, *Journal of Synchrotron Radiation*); however, if you intend to submit to *Acta Crystallographica Section C* or *E* or *IUCrData*, you should make sure that full publication checks are run on the final version of your CIF prior to submission.

Publication of your CIF in other journals

Please refer to the *Notes for Authors* of the relevant journal for any special instructions relating to CIF submission.

PLATON version of 06/07/2023; check.def file version of 30/06/2023

Datablock wsu20-zn - ellipsoid plot

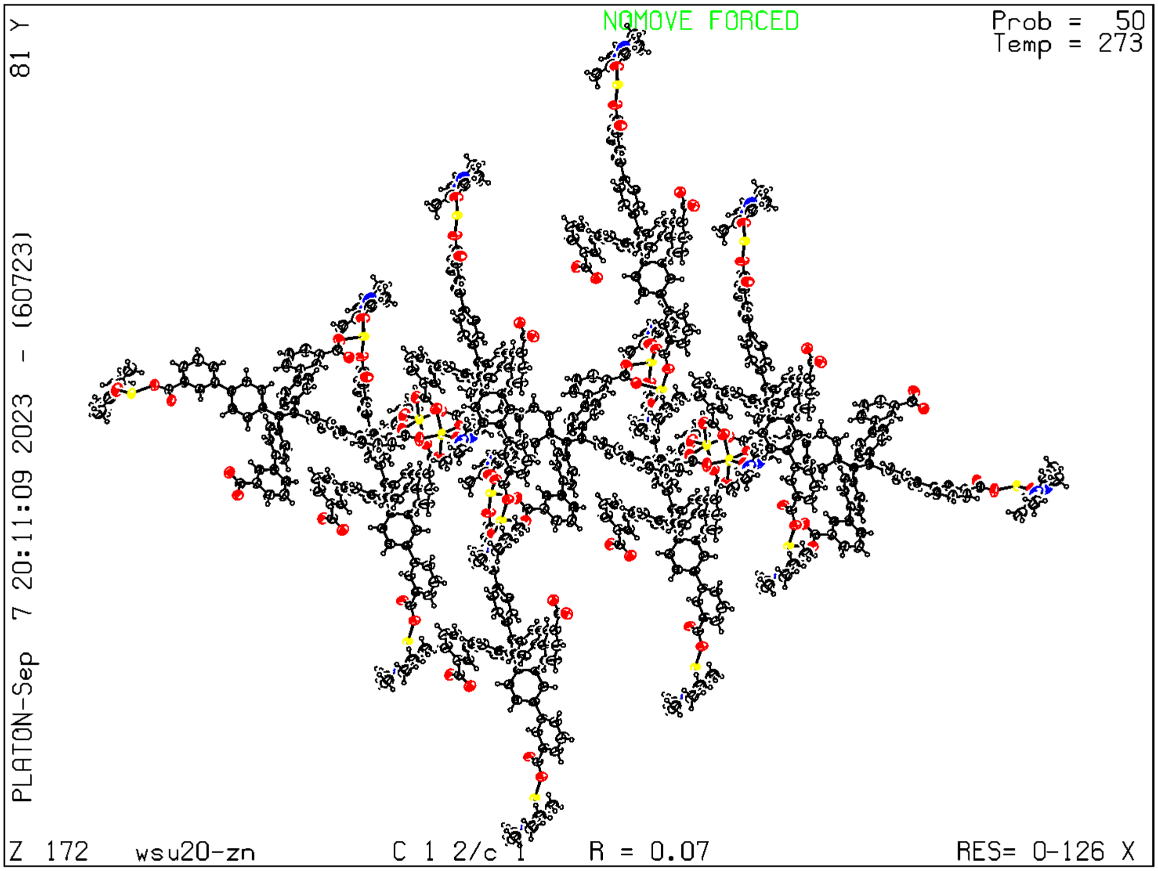

Supplement: Supplementary file 7 [file DataSheet5.PDF]
